# A Systems Approach to Study Immuno- and Neuro-Modulatory Properties of Antiviral Agents

**DOI:** 10.3390/v10080423

**Published:** 2018-08-12

**Authors:** Eva Zusinaite, Aleksandr Ianevski, Diana Niukkanen, Minna M. Poranen, Magnar Bjørås, Jan Egil Afset, Tanel Tenson, Vidya Velagapudi, Andres Merits, Denis E. Kainov

**Affiliations:** 1 Institute of Technology, University of Tartu, 50090 Tartu, Estonia; eva.zusinaite@ut.ee (E.Z.); dianka96-96@mail.ru (D.N.); tanel.tenson@ut.ee (T.T.); andres.merits@ut.ee (A.M.); 2 Norwegian University of Science and Technology (NTNU), 7028 Trondheim, Norway; aleksandr.ianevski@helsinki.fi (A.I.); magnar.bjoras@ntnu.no (M.B.); jan.afset@ntnu.no (J.E.A.); 3 Molecular and Integrative Biosciences Research Programme, Faculty of Biological and Environmental Sciences, University of Helsinki, 00014 Helsinki, Finland; minna.poranen@helsinki.fi; 4 Department of Microbiology, University of Oslo and Oslo University Hospital, 0372 Oslo, Norway; 5 Institute Molecular Medicine Finland (FIMM), University of Helsinki, 00014 Helsinki, Finland; vidya.velagapudi@helsinki.fi

**Keywords:** virus, antiviral agent, drug target, drug side effect, innate immunity, precision medicine, systems biology

## Abstract

There are dozens of approved, investigational and experimental antiviral agents. Many of these agents cause serious side effects, which can only be revealed after drug administration. Identification of the side effects prior to drug administration is challenging. Here we describe an ex vivo approach for studying immuno- and neuro-modulatory properties of antiviral agents, which may be associated with potential side effects of these therapeutics. The current approach combines drug toxicity/efficacy tests and transcriptomics, which is followed by mRNA, cytokine and metabolite profiling. We demonstrated the utility of this approach with several examples of antiviral agents. We also showed that the approach can utilize different immune stimuli and cell types. It can also include other omics techniques, such as genomics and epigenomics, to allow identification of individual markers associated with adverse reactions to antivirals with immuno- and neuro-modulatory properties.

## 1. Introduction

Altogether, 58 known viruses belonging to 20 viral families represent global threats, which are associated with public health concerns and economic burden [1]. Antiviral drugs are powerful tools to control some of the viral diseases. To date, 86 drugs have been approved for the treatment of 17 viral infections [2]. However, some of these agents possess severe side effects [3,4,5]. For example, anti-HCV ribavirin causes hemolytic anemia when used long-term; anti-IAV zanamivir worsens breathing in patients with asthma; and anti-HIV drug, rescriptor, can cause severe rashes and lipodystrophy [6,7,8,9]. The most common side effects for other approved antivirals are nausea, vomiting, allergic reactions, drowsiness, insomnia, behavioral abnormalities, heart problems and dependence (Table S1).

In some cases, the side effects of approved antivirals were discovered during clinical trials and considered as an acceptable risk, whereas, in other cases, the side effects remained unknown until after the drugs were widely used. The latter cases have led to several post-market drug withdrawals, changes in labels, the introduction of new black-box warnings or recommendations [10]. For example, telaprevir and ribavirin were withdrawn after the serious adverse reactions in HCV-infected patients, including skin reactions and birth defects [11]. Amantadine and rimantadine were not recommended for the treatment of IAV infections in 2009, because 100% of the seasonal, as well as the 2009 influenza pandemic strains, carried resistance to the drugs [12]. Amantadine side effects were also associated with the impairment of central nervous system activity [13].

In addition, there are 116 investigational antiviral agents [1]. However, many of these agents have shown severe side effects during phase I, II or III of clinical trials, which prevented them from reaching FDA approval and the market. For example, FDA rejected pleconaril, a drug that prevents common cold symptoms in patients exposed to picornavirus respiratory infections. The decision was based on symptoms such as headache, diarrhea, painful nasal inflammation and nausea as well as emerging drug resistance associated with the treatment [14]. 

Moreover, there are hundreds of experimental antiviral agents [1]. The side effects of many of these agents remain unknown because initial studies have mainly focused on the mechanism of action and therapeutic effects of these compounds. Here, we describe an ex vivo approach to study immuno- and neuro-modulatory properties of prospective antiviral agents. The current approach combines drug toxicity and efficacy tests with genome-wide transcriptomics followed by cytokine and metabolite profiling. We demonstrate the utility of this approach with several examples. Further development of some of these antivirals could be prioritized based on their immuno- and neuro-modulatory profiles.

## 2. On- and Off-Target Side Effects of Antiviral Agents

Antiviral agents can be divided into 2 classes: virus- and host-directed. Most of the approved drugs target viral factors, investigational agents target viruses or host factors, and experimental compounds include mainly host-directed compounds. There will be more discoveries of host-directed antivirals in the future because of increasing research on drug repositioning [15].

Based on the interactions of an antiviral agent with their primary or secondary targets or both, side effects could be classified into three categories [16]. The first category includes on-target side effects, i.e., when an antiviral agent interacts with a host factor, which is critical for virus replication and the viability of infected or non-infected cells. The second category includes off-target side effects, i.e., effects mediated by secondary host targets and pathways, which are not intended to be perturbed by antiviral drugs. The third category includes both on- and off-target effects, i.e., effects, which are associated with the drug’s ability to interact with both primary and secondary targets, which usually belongs to the host. 

Alisporivir is an example of a drug with on-target side effects. It is the most advanced host-directed antiviral in clinical development against HCV. Alisporivir inhibits peptidyl-prolyl isomerase activity of cellular cyclophilin A, which is essential for both viral replication and cell viability. Interaction of alisporivir with cyclophilin A causes hyperbilirubinemia in some patients [17]. 

Anti-HIV rescriptor and anti-IAV amantadine possess off-target side effects [8,18]. Rescriptor inhibits HIV reverse transcriptase and interacts with the histamine H4 receptor. The interaction with H4 receptor causes severe rashes and lipodistrophy. Amantadine targets IAV M2 proton pump and cellular dopamine and adrenalin receptors. The interaction of amantadine with cellular receptors causes nervousness, anxiety, agitation, insomnia, difficulty in concentrating, and exacerbations of pre-existing seizure disorders and psychiatric symptoms in patients with schizophrenia or Parkinson’s disease. 

Broad-spectrum antiviral agents that target cellular protein kinases could possess both on- and off-target side effects. For example, dasatinib, imatinib, and erlotinib target different host kinases, which are needed for efficient virus replication and cell survival [19]. Interaction of these agents with some cellular kinases causes nausea, vomiting, diarrhea, headaches, leg aches/cramps, fluid retention, visual disturbances, itchy rash, bruising, bleeding, loss of appetite, etc. 

**Figure 1:**
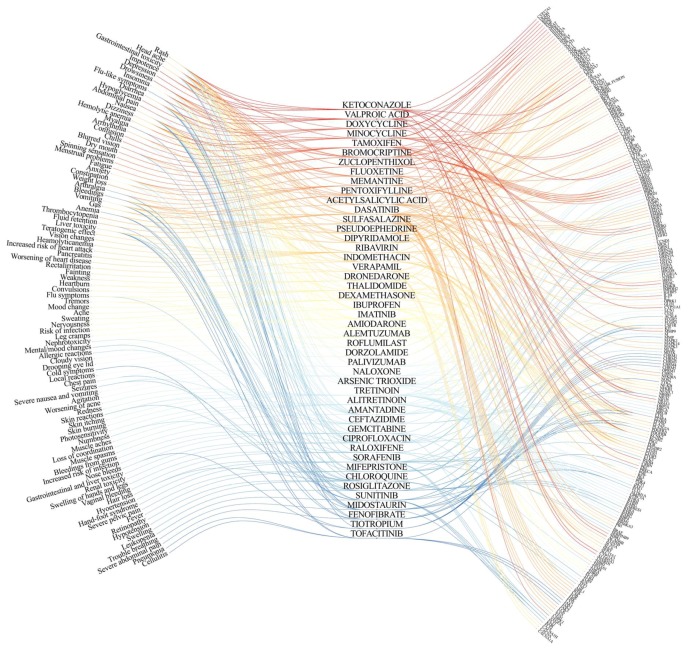
Eye diagram linking antiviral agents (middle) with their side effects (left) and targets (right). The information on drug targets and side effects were retrieved from DrugBank (www.drugbank.ca). Antiviral agents with four or more links to targets or side effects are shown.

Thus, we showed several examples, where the associations of side effects with primary or secondary targets were established. However, the etiology of side effects for many other antiviral agents remain unresolved (Table S1). The future task will be to identify primary and secondary targets for perspective antiviral agents and link them to potential side effects, as in Figure 1.

## 3. Systems Biology Approach to Study Immuno- and Neuro-Modulatory Properties of Antiviral Agents

**Figure 2:**
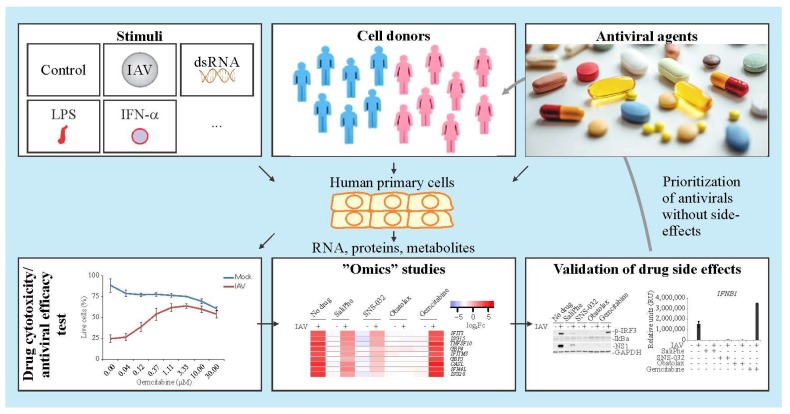
Systems approach to study immuno- and neuro-modulatory properties of antiviral agents.

It is difficult to predict immuno- and neuro-modulatory properties of an antiviral agent. Systems biology approach allows studying such properties in a single experimental setup (Figure 2). It combines drug toxicity/efficacy tests and transcriptomics, which is followed by cytokine and metabolite profiling. In particular, primary patient’s cells are treated with antiviral agents and different immune stimuli. Different omics techniques followed by the integration of the data sets and validation of the results provide novel information about immune- and neuro-modulatory properties of the drugs. The drugs without or with acceptable profiles can be prioritized and given to patients. Thus, the systems biology approach provides a framework to predict the side effects of antiviral drugs based on their immune- and neuro-modulatory profiles and to select appropriate treatment for infected patients.

We have recently utilized a systems biology approach to generate immune- and neuro-modulatory profiles of SaliPhe, SNS-032, obatoclax and gemcitabine [20,21]. These four experimental antivirals inhibit different viruses. In particular, SaliPhe inhibits the endocytic uptake of IAV, FluBV, ZIKV, WNV, JEV, SINV, BUNV, and HPV by targeting cellular vATPase. Obatoclax blocks entry of IAV, IBV, BUNV, SINV, CHIKV, ZIKV, WNV, and YFV by targeting cellular Mcl-1. SNS-032 attenuates IAV and FluBV replication by targeting CDKs. Gemcitabine inhibits transcription and replication of IAV, FluBV, HEV-B, HIV-1, HRV-A, HSV-1, PV, SINV, VACV, and ZIKV by targeting cellular RNR [1,21,22,23,24,25,26]. In addition, all four compounds possess anticancer activity (NCT00446342, NCT00684918) [27,28].

We used PBMC-derived macrophages and different immune stimuli: IAV, dsRNA, bacterial LPS, or IFN-α. DsRNA represents viral PAMPs, LPS is a PAMP of gram-negative bacteria, which could co-infect virus-infected patients, whereas IFN-α is an immuno-mediator, which is produced by infected cells [29]. These stimuli trigger transcription and translation of cellular factors, which are responsible for the production of immune- and neuro-mediators by infected cells. Some of these factors are involved in innate and adaptive immune responses as well as in neurological responses in infected patients.

**Figure 3:**
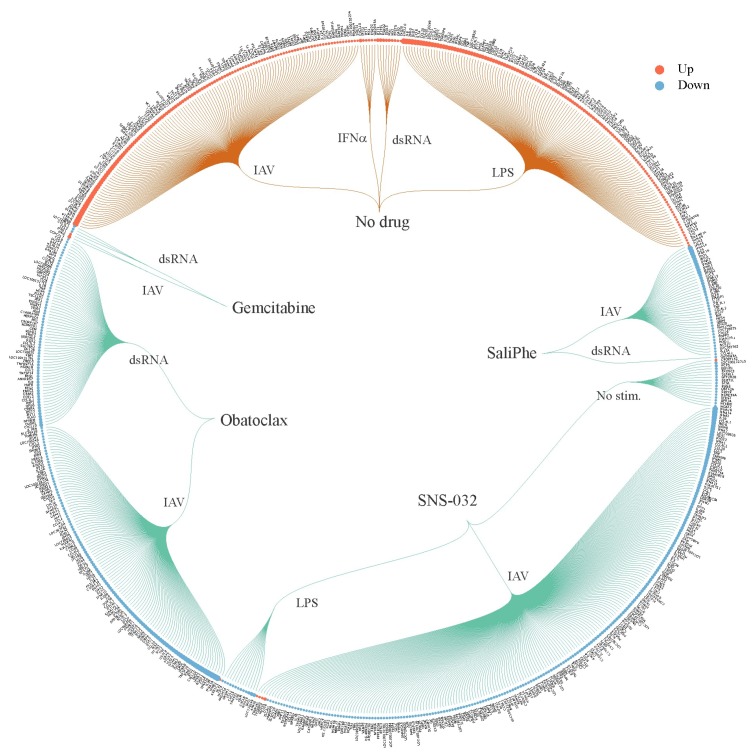
SaliPhe, SNS-032, obatoclax and gemcitabine differentially affect transcription of immune-related genes in stimulated human PBMC-derived macrophages. The macrophages were treated with 3 μM SaliPhe, 0.1 μM SNS-032, 2 μM obatoclax, 1 μM gemcitabine or remained non-treated and infected with IAV (moi 1), or stimulated with 1 μg/mL dsRNA, 1 μg/mL LPS, 1 U/mL IFN-α, or remained non-stimulated. After 8 h, cells were collected; total RNA was extracted and subjected to genome-wide gene expression analysis. Genes, which relative expression levels were up- or down-regulated (log_2_FC_(stimulus_no drug–mock_no drug)_ > 3 and <−3) in response to stimuli in drug non-treated cells, are indicated with orange curves. Genes, which relative expression levels were up- or down-regulated in response to drug treatment in stimulated cells, are shown with green curves (log_2_FC_(stimulus_drug–stimulus_no drug)_ > 3 and <−3). The size of the red and blue circles corresponds to fold changes in expression levels of genes.

We first profiled transcriptional responses of non-/drug-treated resting/stimulated macrophages. We showed that addition of nontoxic but effective concentrations (selectivity index, SI>10) of SaliPhe, SNS-032, obatoclax and gemcitabine to the stimulated cells differentially affected transcription of immune-related genes, including *CCL3, CCL4, CXCL10, IDO1*, and *PTGS2* (Table S2; Figure 3). *CCL3* and *CCL4* encode cytokines, which are involved in the activation of effector cells during immune responses [30,31]. *CXCL10* encodes a cytokine, which attracts monocytes/macrophages, T cells, NK cells, and dendritic cells, and promotes T cell adhesion to endothelial cells. Imbalance of CXCL10 blood levels was associated with psoriasis, cardiovascular and autoimmune diseases [32,33,34]. *IDO1* encodes an enzyme, which catalyzes the first and rate-limiting step in the kynurenine pathway. Dysfunction of the kynurenine pathway was associated with attenuated antigenic immunogenicity, behavior disturbance, and a number of disorders e.g., HIV dementia, Tourette syndrome, tic disorders, psychiatric disorders, multiple sclerosis, Huntington’s disease, encephalopathies, and vitamin B6 deficiency [35,36,37]. PTGS2 (COX2) is responsible for the production of inflammatory prostaglandins. It is a target for NSAIDs, including aspirin and ibuprofen [38].

**Figure 4:**
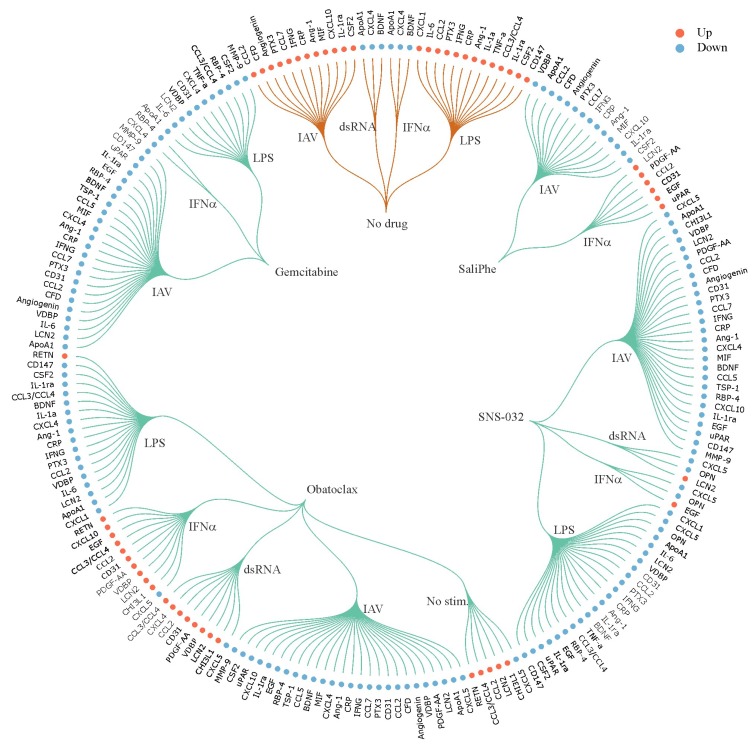
SaliPhe, SNS-032, obatoclax and gemcitabine differentially affect levels of cytokines and growth factors in the culture media of human PBMC-derived macrophages. PBMC-derived macrophages were treated with 3 μM SaliPhe, 0.1 μM SNS-032, 2 μM obatoclax, 1 μM gemcitabine or remained non-treated and infected with IAV (moi 1), or stimulated with 1 μg/mL dsRNA, 1 μg/mL LPS, 1 U/mL IFN-α, or remained non-stimulated. After 24 h, cell culture media were collected and 105 secreted proteins were subjected to analysis with human XL cytokine array kit. Soluble proteins, which relative levels were up- or down-regulated in response to stimuli in the media of non-treated cells, are indicated with orange curves. Soluble proteins, which relative levels were up- or down-regulated in response to stimuli in the media of drug-treated cells, are indicated with green curves.

Next, we used a human cytokine array kit to analyze 105 cytokines and growth factors in the media collected from non-treated/drug-treated resting/stimulated macrophages. We confirmed that SaliPhe, SNS-032, obatoclax and gemcitabine differentially affected the production of cytokines and growth factors by stimulated cells (Table S3; Figure 4).

**Figure 5:**
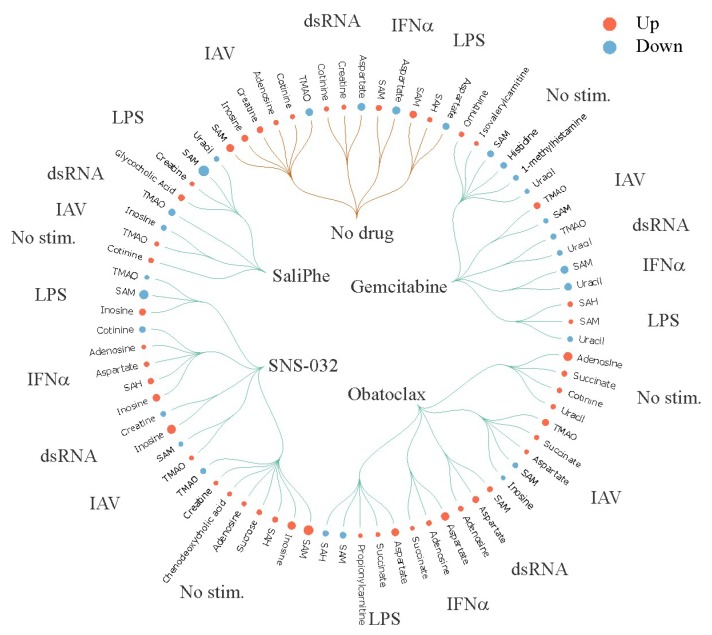
SaliPhe, SNS-032, obatoclax and gemcitabine, differentially affect levels of several polar metabolites in the culture media from stimulated PBMC-derived macrophages. PBMC-derived macrophages were treated with 3 μM SaliPhe, 0.1 μM SNS-032, 2 μM obatoclax, 1 μM gemcitabine or remained non-treated and infected with IAV (moi 1), or stimulated with 1 μg/mL dsRNA, 1 μg/mL LPS, 1 U/mL IFN-α, or remained non-stimulated. After 24 h, cell culture media were collected; polar metabolites were extracted and subjected to targeted metabolomics analysis. Metabolites, which levels were up- or down-regulated (log_2_FC_(stimulus_no drug–mock_no drug)_ > 1.5 and <−1.5) in response to stimuli in the media of drug non-treated cells, are indicated with orange curves. Metabolites, which levels were up- or down-regulated (log_2_FC_(stimulus_drug–stimulus_no drug)_ > 1.5 and <−1.5) in response to drug treatment in the media of activated cells, are shown in green. The size of the red and blue circles corresponds to fold changes in the level of metabolites.

Finally, we analyzed 112 polar metabolites in the media of non-/drug-treated resting/stimulated macrophages. We showed that addition of SaliPhe, SNS-032, obatoclax and gemcitabine to the stimulated cells differentially affected the metabolism of immune- and neuro-modulators, including adenosine and TMAO (Table S4; Figure 5). Adenosine is an anti-inflammatory agent and an inhibitor of the central nervous system [39,40]. TMAO may be involved in the regulation of arterial blood pressure and etiology of hypertension. It was shown that high levels of TMAO in the blood were associated with an increased risk of major adverse cardiovascular events [41].

Thus, we used transcriptomics, proteomics and metabolomics to identified immuno- and neuro-modulatory properties of SaliPhe, SNS-032, obatoclax and gemcitabine in macrophage preparations from different individuals. The identified properties of the compounds are most probably associated with on- and off-target effects, i.e., the agents may target several essential host factors involved in synthesis and metabolism of important immuno- and neuro-modulators. However, it is important to perform follow-up omics analyses on primary cells from the same donor to get consistent results. Moreover, several biological replicates are needed to get meaningful data. Further development of some of these experimental antivirals should not be a priority, because these agents would prevent activation of innate immune and metabolic responses in infected cells that are needed for alarming neighboring cells about ongoing infection and for the protection of the organism from repeated infections. 

## 4. Advantages and Disadvantages of the Approach

The approach can utilize different antiviral agents. We showed recently that JNJ872, which inhibited the transcription and replication of IAV RNA, did not alter cellular antiviral responses at the transcriptional, translational or metabolic levels in human macrophages [42]. By contrast, Akt inhibitor MK2206, which blocks IAV entry, prevented the development of antiviral responses in human non-small-cell lung cancer NCI-H1666 cells [43]. In addition, antiviral Bcl-2 inhibitors including ABT-263 and A-1155463 limited activation of antiviral responses in different cell cultures by inducing the premature death of infected cells [44,45,46]. Thus, the development of JNJ872 should be prioritized because this agent allows development of innate immune and metabolic responses during IAV infection.

Moreover, the current approach can be used to study immune- and neuro-modulatory properties of drugs, which are prescribed for the treatment of underlying diseases in infected patients, such as hypertension, thyroid hormone deficiency and insomnia. This could allow identification of immune- and neuro-modulatory profiles of drugs, the use of which should be omitted in these patients.

The approach can also utilize different cell types such as human monocytes, fibroblasts, epithelial cells of the respiratory or intestinal tract, IPS-derived cell cultures and co-cultures. In our proof-of-concept experiment, we used PBMC-derived macrophages, which differentiation takes 7–10 days. The differentiation step, however, could be omitted, because PBMCs are also susceptible to IAV infection and dsRNA-, IFN-α-, and LPS- stimulation. 

In addition, the approach can use other immune stimuli, including ZIKV, CHIKV, HSV-1, and HIV-1, which infect human monocytes as well as other cell types, which represent natural targets for different viruses [47,48,49,50]. For example, we showed recently that SaliPhe, obatoclax, and gemcitabine affected transcription, translation and posttranslational modifications of cellular factors as well as metabolic pathways in ZIKV-infected human RPE cells [22].

The current approach can also utilize genomics and epigenomics [51,52]. Both techniques were used successfully to link certain viral diseases with genetic variants and epigenetic markers in resting and stimulated monocytes and dendritic cells [29,53,54,55,56,57]. Tying these techniques with transcriptomics, proteomics and metabolomics could allow identification of biomarkers for side effect susceptibility and provide a better understanding of how genetic and epigenetic variations contribute to the efficacy of treatment of viral infections. 

The main disadvantages of the approach, however, is time and costs associated with the experimental setup, omics experiments, data analysis, integration and interpretation. To save time and resources, all analyses should be performed on a single batch of patient cells. In addition, omics studies should be harmonized and target more cytokines (including IFN-β) and metabolites (including prostaglandins). Moreover, the data should be relatively easily annotated, standardized, curated, integrated and interpreted.

## 5. Future Perspectives: Personalized Treatment of Viral Diseases

**Figure 6:**
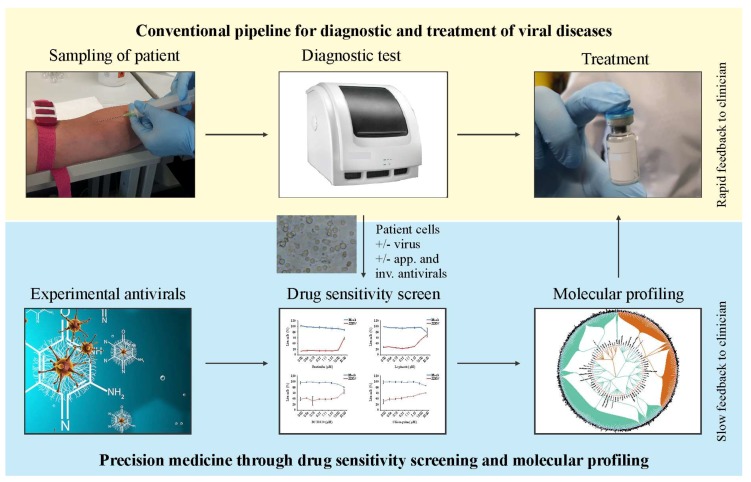
Added value of the systems approach. Systems approaches will guide the evaluation of novel antivirals and their side effects and implementation into personalized medicine pipeline.

To date, many viral infections are diagnosed using PCR- or antibody-based tests. Clinicians evaluate the results of these tests and prescribe treatment for infected patients (Figure 6, upper panel). However, not all patients respond to the standard treatments. Therefore, personalized treatment solutions could become an option for the non-responders [58]. In particular, drug sensitivity screening in resting and infected patient primary cells could identify effective therapeutics among the approved and investigational antiviral agents. This approach, however, will work the best for chronic (such as HCV, HIV, CMV, and HPV), but not acute viral infections, where time is the main constraint. 

Moreover, the number of approved and investigational antivirals is insufficient for the treatment of the entire spectrum of viral infections. This problem could be solved by feeding the drug screens with experimental antiviral compounds, which have been approved for the treatment of non-viral diseases. To our knowledge, there are more than 160 agents with available safety profiles in humans [1]. The drug sensitivity screens followed by molecular profiling of side effects could identify experimental compounds with acceptable immune- and neuro-modulatory effects. Clinicians could assess the screening results, correctly choose between drug options and decide on appropriate dosing and regimen (Figure 6, lower panel).

## 6. Conclusions

There are two sides of the same coin: therapeutic and adverse effects of antivirals. The side effects are usually revealed only after administrating the drugs to patients. Identification of the side effects before drug administration is challenging. Here, we described a systems biology approach, which generates an immune- and neuro-modulatory profile of an antiviral agent using patient cells and different immune stimuli. These profiles allow for the identification of antivirals, which could modulate immune and neurological responses in patients. Based on these profiles, clinicians could prioritize treatment solution for infected patients.

To extract higher value from our study, a harmonized bioactivity data annotation, standardization, curation, and intra-resource integration are needed. We invite other researchers and clinicians to improve the systems biology approach and to test immune- and neuro-modulatory effects of their agents. Tying this approach with patient-specific genetic data and medical records will allow for population-based drug adverse events in cross-sectional studies. Altogether, these studies may decrease mortality of infected patients, maximize the number of healthy life years, and improve the quality of life and cost-effectiveness of patient care.

## Supplementary Materials

The following are available online at http://www.mdpi.com/1999-4915/10/8/423/s1. Table S1: Approved, investigational and experimental antiviral agents and their side effects, Table S2: SaliPhe, SNS-032, obatoclax and gemcitabine differentially affect transcription of immune-related genes in stimulated human PBMC-derived macrophages, Table S3: SaliPhe, SNS-032, obatoclax and gemcitabine differentially affect production of cytokines and growth factors by stimulated human PBMC-derived macrophages, Table S4: SaliPhe, SNS-032, obatoclax and gemcitabine, differentially affect production of several polar metabolites by stimulated PBMC-derived macrophages.

## Abbreviations

PBMC: peripheral blood mononuclear cells; FDA: food and drug administration; IAV: influenza A virus; HIV: human immunodeficiency virus; ZIKV: Zika virus; RPE: retinal pigment epithelium; LPS: lipopolysaccharides; IFN: interferon; dsRNA: double-stranded RNA; SaliPhe: saliphenylhalamide; PAMP: pathogen-associated molecular patterns; TMAO: trimethylamine N-oxide; FluBV: influenza B virus; WNV: West Nile virus; JEV: Japanese encephalitis virus; SINV: Sindbis virus; BUNV: Bunyamwera virus; vATPase: vacuolar ATPase; Mcl-1: induced myeloid leukemia cell differentiation protein; CHIKV: Chikungunya virus; YFV: Yellow fever virus; RNR: ribonucleoside-diphosphate reductase; CDKs: cyclin-dependent protein kinases; NSAIDs: nonsteroidal anti-inflammatory drugs; HPV: human papilloma virus; HEV-B: human enterovirus B; HRV-A: human rhinovirus A; HSV-1: herpes simplex virus 1; PV: poliovirus; VACV: Vaccinia virus.
