# Supplementary material for: A Systems Approach to Study Immuno- and Neuro-Modulatory Properties of Antiviral Agents
Source: Viruses. 2018 Aug 12;10(8):423. doi: 10.3390/v10080423 (PMC6116047; doi:10.3390/v10080423)
Supplement: Supplementary file 1 [file viruses-10-00423-s001.pdf]

Supplementary information

Table S1. Approved, investigational and experimental antiviral agents and their side effects (if known).

Agent	Status	Virus	Potential target	Reference*	Side effects, if known
Abacavir	Approved	HIV-1	HIV-1 RT	DB01048	Allergic reactions, lactic acidosis, liver toxicity, risk of heart attack, nausea, vomiting, lethargy, fever, headache, diarrhea.
		HIV-2	HIV-2 RT		
Aciclovir	Approved	HSV-1	HSV-1 DNA pol	DB00787	Nausea, diarrhea, headache, vomiting, kidney toxicity, low platelets.
		HSV-2	HSV-2 DNA pol		
		VZV	VZV DNA pol		
Adefovir	Approved	HBV	HBV RT	DB13868	Weakness, headache, fever, increased cough, nausea, vomiting, diarrhea, skin rash.
Alitretinoin	Approved	HHV-8	Human RARA	DB00523	Skin reactions, photosensitivity, numbness, muscle aches.
Amantadine	Approved	FLUAV	FLUAV M2	DB00915	Nausea, diarrhea, constipation, headache, insomnia, blurred vision, loss of coordination, rash, confusion.
Amprenavir	Approved	HIV-1	HIV-1 RT	DB00701	Gastrointestinal symptoms (nausea, abdominal pain, diarrhea), paresthesia, depression, rash.
Asunaprevir	Approved	HCV	HCV protease	DB11586	Flu-like symptoms, hair loss, nausea, rash, anemia, clotting problems, liver toxicity.
Atazanavir	Approved	HIV-1	HIV-1 RT	DB01072	Arrhythmia, rash, liver toxicity, flu-like symptoms.
		HIV-2	HIV-2 RT		
BCX4430	Approved	HCV	HCV RNA pol	DB11676	N. a.
Brivudine	Approved	HSV-1	HSV-1 DNA pol	DB03312	Nausea, headache, lowered blood cell counts, liver toxicity, allergic reactions.
		VZV	VZV DNA pol		
Cidofovir	Approved	CMV	CMV DNA pol	DB00369	Headache, nausea, vomiting, abdominal pain, rash, liver toxicity.
Cobicistat	Approved	HIV-1	HIV-1 integrase	DB09065	Nausea, dizziness, liver and kidney toxicity,
		HIV-2	HIV-2 integrase		
Daclatasvir	Approved	HCV	HCV NS5A	DB09102	Headache, nausea, diarrhea, insomnia, rash, anemia.
Darunavir	Approved	HIV-1	HIV-1 protease	DB01264	Diarrhea, nausea, rash, headache, stomach pain, vomiting, liver toxicity.
Dasabuvir	Approved	HCV	HCV RNA pol	DB09183	Weakness, rash, liver toxicity, anemia.
Delavirdine	Approved	HIV-1	HIV-1 RT	DB00705	Nausea, diarrhea, headache, changes in body fat, flu-like symptoms.
Didanosine	Approved	HIV-1	HIV-1 RT	DB00900	Headache, diarrhea, rash, changes in body fat, liver toxicity, seizures.
		HIV-2	HIV-2 RT		
Docosanol	Approved	HSV-1	HSV-1 glycoproteins	DB00632	Allergic reactions, rash, headache.
		HSV-2	HSV-2 glycoproteins		
Dolutegravir	Approved	HIV-1	HIV-1 integrase	DB08930	Insomnia, fatigue, headache, liver toxicity.
		HIV-2	HIV-2 integrase		
Dronabinol	Approved	HIV-1	Human CNR1	DB00470	Seizures, anxiety, arrhythmia, dizziness, lack of coordination, insomnia, gastrointestinal toxicity.

Efavirenz	Approved	HIV-1	HIV-1 RT	DB00625	Mental health problems, liver toxicity, severe rash, birth defects in animals.
Elbasvir	Approved	HCV	HCV NS5A	DB11574	Fatigue, headache, gastrointestinal symptoms, rash, arthralgia, anemia.
Elvitegravir	Approved	HIV-1	HIV-1 integrase	DB09101	Alterations of immune system, diarrhea, nausea, rash, gastrointestinal toxicity.
		HIV-2	HIV-2 integrase		
Emtricitabine	Approved	HIV-1	HIV-1 RT	DB00879	Flu-like symptoms, paresthesia, arrhythmia, gastrointestinal toxicity, liver toxicity, insomnia.
	Approved	HIV-2	HIV-2 RT		
Enfuvirtide	Approved	HIV-1	HIV-1 gp41	DB00109	Local reactions, numbness, insomnia, depression, arthralgia, arrhythmia, vision changes, flu-like symptoms, Guillain-Barre Syndrome.
Entecavir	Approved	HBV	HBV DNA pol	DB00442	Lliver toxicity, lactic acidosis, arrhythmia, gastrointestinal toxicity, hair loss, insomnia.
Etravirine	Approved	HIV-1	HIV-1 RT	DB06414	Severe skin rash, allergic reactions, arthralgia, liver toxicity, gastrointestinal toxicity.
Famciclovir	Approved	HSV-1	HSV-1 DNA pol	DB00426	Headache, nausea, diarrhea, liver and kidney toxicity, mental/mood changes.
		HSV-2	HSV-2 DNA pol		
		VZV	VZV DNA pol		
Favipiravir	Approved	FLUAV	FLUBV RNA pol	DB12466	N. a.
		FLUCV	FLUCV RNA pol		
		FLUBV	FLUAV RNA pol		
Fomivirsen	Approved	CMV	CMV mRNA UL123	DB06759	Eye inflammation, vision abnormalities, cataracts, retinal detachment, anemia, flu-like symptoms, rash
Fosamprenavir	Approved	HIV-1	HIV-1 protease	DB01319	Severe skin reactions, allergic reactions, gastrointestinal symptoms, headache.
Foscarnet	Approved	CMV	CMV DNA pol	DB00529	Headache, gastrointestinal toxicity, mental/mood changes, arrhythmia, muscle cramps.
		HSV-1	HSV-1 DNA pol		
		HSV-2	HSV-2 DNA pol		
Ganciclovir	Approved	CMV	CMV DNA pol	DB01004	Local reactions, gastrointestinal toxicity, tremors, infertility.
Glecaprevir	Approved	HCV	HCV protease	DB13879	Diarrhea, headache, nausea, itching skin, weakness.
Grazoprevir	Approved	HCV	HCV protease	DB11575	Headache, gastrointestinal toxicity, rash, arthralgia, depression, anemia.
HBIG	Approved	HBV	HBV proteins	DB05276	Flu-like symptoms, back pain, gastrointestinal symptoms, serious allergic reactions, fever, liver toxicity, risk of stroke.
Idoxuridine	Approved	HSV-1	HSV-1 DNA pol	DB00249	Eye irritation, pain, swelling, increased sensitivity to light.
Imiquimod	Approved	HPV	Human TLR7	DB00724	Local skin reactions, headache, flu-like symptoms, chest and back pain, gastrointestinal symptoms.
IMOGAM	Approved	RABV	RABV proteins	DB11603	Headache, flu-like symptoms, gastrointestinal symptoms, rash, allergic reactions.
Indinavir	Approved	HIV-1	HIV-1 protease	DB00224	Kidney and liver toxicity, hemolytic anemia, gastrointestinal toxicity, arthralgia, thyroid toxicity.
		HIV-2	HIV-2 protease		

Interferon alfacon 1	Approved	HCV	Human IFNAR1	DB00069	Mood changes, headache, gastrointestinal symptoms, arthralgia.
Lamivudine	Approved	HBV	HBV RT	DB00709	Headache, gstrointestinal and liver toxicity, rash, pancreatitis, anemia, fat redistribution.
		HIV-1	HIV-1 RT		
		HIV-2	HIV-2 RT		
Laninamivir octanoate	Approved	FLUBV	FLUBV NA	DB11888	Psychiatric disorders, gastrointestinal symptoms, dizziness.
		FLUAV	FLUAV NA		
Ledipasvir	Approved	HCV	HCV NS5A	DB09027	Fatigue, headache, nausea, diarrhea, insomnia.
Letermovir	Approved	CMV	CMV TRM1	DB12070	Cough, headache, gastrointestinal toxicity.
Lopinavir	Approved	HIV-1	HIV-1 protease	DB01601	Headache, fat redistribution, gastrointestinal symptoms, liver toxicity, allergic reactions, flu-like symptoms, insomnia.
		HIV-2	HIV-2 protease		
Maraviroc	Approved	HIV-1	Human CCR5	DB04835	Liver toxicity, skin rash, allergic reactions, flu-like symptoms.
		HIV-2	Human CCR5		
Nelfinavir	Approved	HIV-1	HIV-1 protease	DB00220	Allergic reactions, confusion, high blood sugar, bleedings, risk of infections and autoimmune disorders.
		HIV-2	HIV-2 protease		
Nevirapine	Approved	HIV-1	HIV-1 RT	DB00238	Gastrointestinal toxicity, muscle pain, headache, body fat redistribution.
Ombitasvir	Approved	HCV	HCV NS5A	DB09296	Nausea, waekness, insomnia, allergic reactions.
Oseltamivir	Approved	FLUBV	FLUBV NA	DB00198	Headache, gastrointestinal symptoms, insomnia, nosebleed, mental/mood changes.
		FLUAV	FLUAV NA		
Palivizumab	Approved	RSV	RSV F	DB00110	Cold symptoms, gastrointestinal symptoms, rash, local reactions.
Paritaprevir	Approved	HCV	HCV protease	DB09297	Fatigue, skin reactions, insomnia, weakness.
PegIFNα-2a	Approved	HBV	Human IFNAR1	DB00008	Flu-like symptoms, anemia, leukopenia.
		HCV	Human IFNAR1		
PegIFNα-2b	Approved	HBV	Human IFNAR1	DB00022	Leukopenia, thyroid dysfunction.
		HCV	Human IFNAR1		
Penciclovir	Approved	HSV-1	HSV-1 DNA pol	DB00299	Application site reactions, headache, allergic reactions
		HSV-2	HSV-2 DNA pol		
Peramivir	Approved	FLUBV	FLUBV NA	DB06614	Diarrhea, constipation, insomnia, high blood pressure, allergic reactions.
		FLUAV	FLUAV NA		
Pibrentasvir	Approved	HCV	HCV NS5A	DB13878	Headache, nausea, diarrhea, itching.
Podofilox	Approved	HPV	Human TOP2A	DB01179	Skin reactions, insomnia, nausea, vomiting.
Raltegravir	Approved	HIV-1	HIV-1 integrase	DB06817	Gastrointestinal toxicity, insomnia, body fat redistribution.
		HIV-2	HIV-2 integrase		
Ribavirin	Approved	HCV	HCV RNA pol	DB00811	Hemolytic anemia, increased risk of heart attack, arrhythmia, teratogenic effect.
		RSV	RSV RNA pol		
Rilpivirine	Approved	HIV-1	HIV-1 RT	DB08864	Severe rash, allergic reactions, liver toxicity, depression, body fat redeistribution, insomnia.

Rimantadine	Approved	FLUAV	FLUAV M2	DB00478	Insomnia, allergic reactions, mental/mood changes, seizures.
Ritonavir	Approved	HIV-1	HIV-1 protease	DB00503	Gastrointestinal symptoms, headache, Guillain-Barre syndrome, thyroid toxicity, alterations of immune system.
		HIV-2	HIV-2 protease		
RSV-IGIV (RespiGam)	Approved	RSV	RSV F	N.A.	Nausea, local reactions, myalgia/arthralgia, liver toxicity, allergic reactions, aseptic meningitis.
Saquinavir	Approved	HIV-1	HIV-1 protease	DB01232	Gastrointestinal toxicity, body fat redistribution, arrhythmia, liver toxicity.
		HIV-2	HIV-2 protease		
Simeprevir	Approved	HCV	HCV protease	DB06290	Rash, sun sensitivity, nausea, myalgia.
Sinecatechins	Approved	HPV	Unknown	DB01266	Local skin reactions, allergic reactions.
Sofosbuvir	Approved	HCV	HCV RNA pol	DB08934	Nausea, diarrhea, headache, anemia, allergic reactions.
Stavudine	Approved	HIV-1	HIV-1 RT	DB00649	Headache, diarrhea, myalgia/arthralgia, vision changes, thyroid toxicity, Guillain-Barre syndrome.
Telaprevir	Approved	HCV	HCV protease	DB05521	Allergic reaction, skin reactions, gastrointestinal toxicity.
Telbivudine	Approved	HBV	HBV RT	DB01265	Dizziness, diarrhea, headache, allergic reactions.
Tenofovir alafenamide	Approved	HBV	HBV RT	DB09299	Abdominal pain, nausea, osteoporosis, pneumonia.
Tenofovir disoproxil	Approved	HBV	HBV RT	DB00300	Gastrointestinal symptoms, mental/mood changes, rash.
		HIV-1	HIV-1 RT		
		HIV-2	HIV-2 RT		
Thymalfasin	Approved	HBV	Unknown	DB04900	Muscle atrophy, arthralgia, rash, local reactions.
		HCV	Unknown		
Tipranavir	Approved	HIV-1	HIV-1 protease	DB00932	Liver toxicity, risk of stroke, gastrointestinal toxicity, body fat redistribution.
Trifluridine	Approved	HSV-1	HSV-1 DNA pol	DB00432	Eye irritation, blurred vision, allergic reactions.
		HSV-2	HSV-2 DNA pol		
		VACV	VACV DNA pol		
Valacyclovir	Approved	CMV	CMV DNA pol	DB00577	Headache, mental/mood changes, kidney toxicity, allergic reactions.
		EBV	EBV DNA pol		
		HBV	HBV DNA pol		
		HSV-1	HSV-1 DNA pol		
		HSV-2	HSV-2 DNA pol		
		VZV	VZV DNA pol		
Valganciclovir	Approved	CMV	CMV DNA pol	DB01610	Diarrhea, kidney toxicity, mental/mood changes, allergic reactions, seizures.
VariZIG	Approved	VZV	VZV proteins	DB11621	Flu-like symptoms, local reactions, allergic reaction, arthralgia.
Velpatasvir	Approved	HCV	HCV NS5A	DB11613	Headache, insomnia, anemia, rash, depression.
Vidarabine	Approved	HSV-1	HSV-1 DNA pol	DB00194	Lacrimation, photophobia, keratitis, irritation.
		HSV-2	HSV-2 DNA pol		
		VZV	VZV DNA pol		

Voxilaprevir	Approved	HCV	HCV protease	DB12026	Allergic reactions, liver toxicity, diarrhea.
Zalcitabine	Approved	HIV-1	HIV-1 RT	DB00943	Constipation, diarrhea, headache, body fat redistribution, convulsions, allergic reactions.
		HIV-2	HIV-2 RT		
Zanamivir	Approved	FLUBV	FLUBV NA	DB00558	Dizziness, allergic reactions.
		FLUAV	FLUAV NA		
Zidovudine	Approved	HIV-1	HIV-1 RT	DB00495	Headache, nausea, insomnia, arthralgia, thyroid toxicity, Guillain-Barre syndrome.
		HIV-2	HIV-2 RT		
ABI-H0731	Investigational	HBV	HBV Cp	NCT03109730	N. a.
ABX464	Investigational	HIV-1	HIV-1 RT	NCT02990325	Headache, nausea, vomiting.
		HIV-2	HIV-2 RT	NCT02990325	
ACH-2684	Investigational	HCV	HCV protease	N.A.	N. a.
ACH-3422	Investigational	HCV	HCV RNA pol	N.A.	N. a.
AK0529	Investigational	RSV	RSV F	NCT02654171	N. a.
Albuterpenoid	Investigational	MCV	Unknown	N.A.	N. a.
ALN-HBV	Investigational	HBV	HBV mRNA	NCT02826018	N. a.
Alisporivir	Investigational	HCV	Human PPIA	NCT02094443	Nausea, headache, liver toxicity.
ALS-008176	Investigational	RSV	RSV RNA pol	NCT02673476	Epistaxis, cough.
ALS-8112	Investigational	RSV	RSV RNA pol	N.A.	N. a.
ALX-0171	Investigational	RSV	RSV F	NCT02979431	N. a.
APH-0812	Investigational	HIV-1	Human PKC	N.A.	N. a.
Aprepitant	Investigational	HIV-1	Human TAKR1	NCT02154360	Gastrointestinal toxicity, insomnia, headache, flu-like symptoms.
ARB-1467 (ARB-001467)	Investigational	HBV	HBV transcripts	NCT02631096	N. a.
Astodrimer	Investigational	HIV-1	HIV-1 gp120	NCT00740584	Genitourinary symptoms, bagterial vaginosis, bleeding between menstrual periods.
		HSV-2	HSV-2 DNA pol	NCT00740584	
AV4025	Investigational	HCV	HCV NS5A	N.A.	N. a.
AVI-7100	Investigational	FLUAV	FLUAV M1/M2 mRNA	NCT01747148	Headache, proteinuria.
AVI-7288	Investigational	MARV	MARV NP mRNA	NCT01566877	N. a.
AVI-7537	Investigational	EBOV	EBOV VP24 mRNA	NCT01593072	Headache, nausea, fatigue.
AVR560	Investigational	HCV	Unknown	N.A.	N. a.
Bavituximab	Investigational	HCV	Human Ptd-L-Ser	NCT01273948	Anemia, neutropenia, fatigue.
		HIV-1	Human Ptd-L-Ser	NCT00503347	
BCX4430	Investigational	EBOV	EBOV RNA pol	NCT02319772	N. a.
		HCV	Human PPIA	N.A.	
		MARV	MARV RNA pol	N.A.	
Beraprost	Investigational	FLUAV	Human CYP2C8	N.A.	Headache, diarrhea.
Bictegravir	Investigational	HIV-1	HIV-1 integrase	NCT02603107	Headache, nausea, diarrhea, insomnia.
Birinapant	Investigational	HBV	Human IAP	NCT02288208	N. a.
BIT225	Investigational	HCV	HCV p7	N.A.	N. a.
BL-8020	Investigational	HCV	Unknown	N.A.	N. a.
BMS-955176	Investigational	HIV-1	HIV-1 GAG	NCT01803074	Headache, liver toxicity, diarrhea.

Brincidofovir	Investigational	AdV	AdV DNA pol	NCT02087306	Renal toxicity, abdominal cramps, diarrhea.
		BKV	Unknown	NCT01769170	
		CMV	CMV DNA pol	NCT01769170	
		EBV	EBV DNA pol	NCT01769170	
		HHV-6	HHV-6 DNA pol	NCT01769170	
BTA074 (AP611074)	Investigational	HPV	HPV E1 and E2	NCT02724254	N. a.
BTL-TML-001	Investigational	FLUAV	Unknown	N.A.	N. a.
BTL-TML-HSV	Investigational	HSV-1	Unknown	NCT01902303	N. a.
Cabotegravir	Investigational	HIV-1	HIV-1 integrase	NCT02951052	Headache, flu-like symptoms, diarrhea.
CC-31244	Investigational	HCV	HCV RNA pol	NCT02760758	N. a.
Cenicriviroc	Investigational	HIV-1	Human CCR5	NCT01338883	Nausea, headache, dearrhea.
Cidofovir	Investigational	BKV	BKV DNA pol	NCT03216967	Liver and gastrointestinal toxicity, vision changes, allergic reactions.
		CMV	CMV DNA pol	NCT00002437	
		HPV	HPV DNA pol	NCT00550589	
		HSV-1	HSV-1 DNA pol	NCT01431326	
		HSV-2	HSV-2 DNA pol	NCT01431326	
Ciprofloxacin	Investigational	BKV	Human TOP2A	NCT01789203	Headache, insomnia, gastrointestinal and liver toxicity, renal toxicity, allergic
Civacir	Investigational	HCV	HCV proteins	NCT01804829	Flu-like syndrome, allergic reactions.
Clemizole	Investigational	HCV	HCV NS4B	NCT00945880	N. a.
Chloroquine	Investigational	FLUAV	Human TNF	NCT01078779	Gastrointestinal toxicity, mental/mood changes, liver toxicity, bleedings.
Cyclopropavir	Investigational	CMV	CMV UL97	NCT02454699	N. a.
CYT107	Investigational	HBV	Human CD127	NCT01027065	Liver toxicity, rash, local reactions, flu-like symptoms.
		HCV	Human CD127	NCT01025596	
		HIV-1	Human CD127	NCT01019551	
Cytolin	Investigational	HIV-1	Human CD11a	N.A.	N. a.
Danoprevir	Investigational	HCV	HCV protease	NCT03020082	Flu-like syndrome, headache, anemia, neutropenia.
DAS181	Investigational	FLUAV	Human SA	NCT01037205	Increase of alkaline phosphatase level
Doravirine	Investigational	HIV-1	HIV-1 RT	NCT02403674	Nausea, depression, diarrhea, insomnia.
EDP-239	Investigational	HCV	HCV NS5A	NCT01856426	N. a.
EDP-494	Investigational	HCV	Human PPIA	NCT02652377	N. a.
Emricasan	Investigational	HCV	Human CASP1	NCT00088140	Headache, nausea, fatigue.
Enzaplatovir (BTA585)	Investigational	RSV	RSV F	NCT02718937	N. a.
Everolimus	Investigational	BKV	Human MTOR	NCT01624948	Gastrointestinal and liver toxicity, headache, arthralgia, myalgia.
		HCV	Human MTOR	NCT01134952	
EYP001	Investigational	HBV	Human IFNAR1	NCT03272009	Gastrointestinal symptoms, headache, muscular cramps.
Faldaprevir	Investigational	HCV	HCV protease	NCT01297270	Gastrointestinal symptoms, anemia, rash.
Favipiravir	Investigational	EBOV	EBOV RNA pol	NCT02329054	N. a.
Fostemsavir (BMS-663068)	Investigational	HIV-1	HIV-1 GP120	NCT02362503	Headache, rash, gastrointestinal toxicity.
FV-100	Investigational	VZV	VZV DNA pol	NCT02412917	Gastrointestinal toxicity, headache, trembling.

GS-5734	Investigational	EBOV	EBOV RNA pol	NCT02818582	N. a.
GS-5806	Investigational	RSV	RSV F	NCT02135614	N. a.
GSK3228836 (IONIS HBVRx)	Investigational	HBV	HBV RNA	NCT02981602	N. a.
GSK3389404	Investigational	HBV	HBV RNA	NCT03020745	N. a.
Ibalizumab	Investigational	HIV-1	Human CD4	NCT02475629	Diarrhea, anemia, rash, liver toxicity.
IDX375	Investigational	HCV	HCV RNA pol	N.A.	N. a.
IFN-alpha 2B-XL	Investigational	HBV	Human IFNAR1	N.A.	Flu-like symptoms, liver toxicity, arrhythmia, nausea, allergic reactions.
		HCV	Human IFNAR1	NCT01010646	
IQP-0528	Investigational	HIV-1	HIV-1 RT	NCT03082690	N. a.
JNJ-56914845 (GSK2336805)	Investigational	HCV	HCV NS5A	NCT01439373	N. a.
Lomibuvir (VX-222)	Investigational	HCV	HCV protease	NCT01516918	N. a.
Lonafarnib	Investigational	HDV	Human FTI	NCT02511431	Diarrhea, nausea, weight loss.
Maribavir	Investigational	CMV	CMV DNA pol	NCT00497796	Taste disturbances, diarrhea, rash, anemia.
MDT-637	Investigational	RSV	RSV F	NCT01355016	N. a.
MEDI8852	Investigational	FLUAV	FLUAV HA	NCT03028909	Headache
MEDI8897	Investigational	RSV	RSV F	NCT02878330	Headache, dermatitis.
Mericitabine	Investigational	HCV	HCV RNA pol	NCT01482390	Headache, nausea, diarrhea, flu-like syndrome, insomnia, dizziness.
Merimepodib	Investigational	HCV	Human IMPDH2	NCT00088504	Nausea, arthralgia, dyspnea, neutropenia, anemia.
Miravirsen	Investigational	HCV	Human miR-122	NCT01200420	N. a.
MK-3682	Investigational	HCV	HCV RNA pol	NCT02332720	Headache, nausea, fatigue.
Mycophenolic acid	Investigational	BKV	Human IMPDH2	NCT01624948	Gastrointestinal toxicity, insomnia, myalgia, headache.
Myrcludex B	Investigational	HBV	HBV pre-S1	NCT02888106	Local reactions, increased liver enzymes level.
Namodenoson (CF-102)	Investigational	HCV	Human A3AR	NCT00790673	N. a.
Nitazoxanide	Investigational	FLUAV	FLUAV HA	NCT02612922	Headache, gastrointestinal symptoms.
		HCV	Human PKR	NCT01770483	
N-Methanocarbathymidine	Investigational	HSV-2	HSV-2 DNA pol	N.A.	N. a.
Odalasvir	Investigational	HCV	HCV NS5A	NCT03099135	Headache, fatigue, upper respiratory tract infections.
OHR118	Investigational	HIV-1	Human ACVR2B	N.A.	N. a.
		HPV	Human ACVR2B	N.A.	
Paritaprevir	Investigational	HCV	HCV protease	NCT02992457	Rash, insomnia, allergic reactions.
PEG-Interferon lambda	Investigational	HCV	Human IL28R	NCT01598090	Elevated liver enzymes.
PEG-P-IFN alpha-2b	Investigational	HCV	Human IFNAR1	NCT00351871	Headache, hematologic abnormalities, flu-like syndrome.
Pimodivir	Investigational	FLUAV	FLUAV RNA pol	NCT03376321	Diarrhea, nausea.
Polyacrylic acid	Investigational	HIV-1	HIV-1 Tat	NCT00446979	Blurred vision, burning.
PPI-461	Investigational	HCV	HCV NS5A	NCT01247194	N. a.
Pritelivir	Investigational	HSV-1	HSV-1 helicase-primase	NCT03073967	Local skin reactions.
		HSV-2	HSV-2 helicase-primase	NCT01047540	

Rapamycin	Investigational	CMV	Human MTOR	NCT02683291	Thrombocytopenia, nephrotoxicity, altered insulin sensitivity.
Ravidasvir	Investigational	HCV	HCV NS5A	NCT03362814	Headache, diarrhea, itching.
RDEA806	Discontinued	HIV-1	HIV-1 RT	NCT00617526	N. a.
REGN2222	Investigational	RSV	RSV F	NCT02325791	Injection site reactions, allergic reactions.
REP2139	Investigational	HBV	Unknown	NCT02233075	N. a.
Rifamycin	Investigational	HCV	Human NR1I2	N.A.	Gastrointestinal and liver toxicity, headache, drowsiness, heartburn.
		HIV-1	Human NR1I2	N.A.	
Rintatolimod (ampligen)	Investigational	FLUAV	Human TLR3	NCT01591473	Flu-like symptoms, flushing.
		HIV-1	Human TLR3	NCT00035893	
RV521	Investigational	RSV	RSV F	NCT03258502	N. a.
S-033188 (baloxavir marboxil)	Investigational	FLUAV	FLUAV RNA pol	NCT02954354	Headache, diarrhea, increased liver enzymes
Samatasvir	Investigational	HCV	HCV NS5A	NCT01852604	N. a.
SB 9200	Investigational	HBV	Human DDX58	NCT02751996	N. a.
		HCV	Human DDX58	NCT01803308	
SB-728-T	Investigational	HIV-1	Human CCR5	NCT01543152	Infusion-related reactions.
SCY-635	Investigational	HCV	Human PPIA	NCT01265511	N. a.
SD-101	Investigational	HCV	Human TLR9	NCT00823862	N. a.
Simvastatin	Investigational	HCV	Human HMGCR	NCT01377909	Memory problems, diabetes, liver toxicity, allergic reactions.
Sorivudine	Investigational	VZV	VZV DNA pol	NCT00002358	N. a.
TD-6450	Investigational	HCV	HCV NS5A	NCT02593162	N. a.
Tenofovir disoproxil	Investigational	HSV-2	HSV-2 DNA pol	NCT01386294	Lactic acidosis, liver toxicity,
Thymalfasin	Investigational	HIV-1	Unknown	NCT00001036	Depression, insomnia, body fat redistribution, gastrointestinal toxicity.
TMC278	Investigational	HIV-1	HIV-1 RT	NCT01777997	Gastrointestinal symptoms, rash, depression, body fat redistribution, insomina.
TMC647055	Investigational	HCV	HCV RNA pol	NCT01724086	N. a.
Traumakine	Investigational	FLUAV	Human IFNAR1	N.A.	N. a.
UV-4B	Investigational	DENV	Human glucosidases	NCT02061358	Osmotic diarrhea.
		EBOV	Human glucosidases	N.A.	
VIS410	Investigational	FLUAV	FLUAV HA	NCT03040141	Diarrhea, headache.
VX-135	Investigational	HCV	HCV RNA pol	NCT01726946	Fatigue, headache, nausea.
Acetaminophen	Experimental	DENV	DENV NS4B	26856827	Allergic reactions, rash, dizziness.
Acetylsalicylic acid	Experimental	HCV	Human PTGS1	26289738	Rash, gastrointestinal toxicity, drowsiness, bleedings.
		HSV-1	Human PTGS1	24768597	
		FLUAV	Human PTGS1	17324159	
Alemtuzumab	Experimental	HIV-1	Human CD52	27482429	Nausea, dizziness, vomiting, headache, sweating, mental/mood changes, nephrotoxicity, liver toxicity.
Alisporivir	Experimental	HIV-1	Human PPIA	19857522	N. a.
		MERS-CoV	Human PPIA	27840112	

Amiodarone	Experimental	EBOV	Human KCNH2	25933611	Nausea, vomiting, constipation, tremor, insomnia, headache.
Anakinra	Experimental	FLUAV	Human IL1R1	28740490	Worsening of rheumatoid arthritis symptoms, nausea, diarrhea, headache, local reactions.
Arsenic trioxide	Experimental	EBV	Human IKBKB	28637474	Gastrointestinal toxicity, arrhythmia, rash, arthralgia, insomnia.
		HPV	Human IKBKB	25117446	
Artemether	Experimental	EBOV	Human CYP3A4	26735991	Diarrhea, headache, nausea, dizziness, fainting, rash, insomina, arthralgia, myalgia.
Atorvastatin	Experimental	DENV	Human HMGCR	27431377	Myalgia, confusion, memory problems, fever, fluid retension, liver toxicity.
Azacitidine	Experimental	AdV	AdV DNA pol	11562975	Nausea, anemia, thrombocytopenia, leukopenia, fever, diarrhea, rash.
		FLUAV	FLUAV RNA pol	11562975	
		HIV-1	HIV-1 RT	27117260	
		HIV-2	HIV-2 RT	25103850	
Azelastine	Experimental	HBV	Human NTCP	24965018	Drowsiness, nose bleeds, nausea, dry mouth, weight gain, headache, myalgia.
Azithromycin	Experimental	EBOV	Human PADI4	27622822	Gastrointestinal symptoms, liver toxicity, allergic reactions, arrhythmia.
		HRV-A	Human PADI4	28415826	
		ZIKV	Human PADI4	27911847	
BCX4430	Experimental	RAVV	RAVV RNA pol	24590073	N. A.
		TBEV	TBEV RNA pol	28336346	
		WNV	WNV RNA pol	28336346	
		YFV	YFV RNA pol	25155605	
		ZIKV	ZIKV RNA pol	27838352	
Bepotastine besilate	Experimental	EMCV	Human HRH1	18606918	Headache, eye irritation, allergic reactions.
Betamethasone	Experimental	FLUAV	Human CYP3A4	21168053	Acne, dry skin, insomnia, sweating, headache, nausea.
Bortezomib	Experimental	DENV	Human PSMB5	26565697	Gastrointestinal toxicivy, arthralgia, myalgia, insomina, rash.
		HBV	Human PSMB5	19949053	
		FLUAV	Human PSMB5	20592098	
		RSV	Human PSMB5	19931343	
		RVFV	Human PSMB5	26001632	
		VSV	Human PSMB5	20592098	
		ZIKV	Human PSMB5	27476412	
Bosentan	Experimental	HEV-B	Human EDNRB	20392896	Nausea, liver toxicity, headache, flushing, itching.
Bromocriptine	Experimental	DENV	DENV protease-helicase	27181378	Drowsiness, spinning sensation, headache, insomnia, dry mouth, gastrointestinal symptoms.
		ZIKV	ZIKV protease	28185815	
Budesonide	Experimental	HRV-B	Human CYP3A4	24998372	Headache, sore throat, nausea, rash, myalgia.
		HRV-A	Human CYP3A4	24219422	
Cabozantinib	Experimental	ZIKV	Human MET	27650556	Insomnia, nervousness, stomach irritation, nausea, vomiting, increased heart rate.
Caffeine	Experimental	HCV	Human ADORA1	25491197	

		HSV-1	Human ADORA1	21725588	
		FLUAV	Human ADORA1	25050906	
		JV	Human ADORA1	10509392	
		VZV	Human ADORA1	6257827	
Capsaicin	Experimental	HSV-2	Human TRPV1	10543747	Skin redness, itching, burning, pain.
Ceftazidime	Experimental	HIV-1	Human SLC22A5	11527042	Muscle spasms, seizures, nephrotoxicity, liver toxicity, abdominal pain.
Chloroquine	Experimental	CHIKV	Human TNF	21503920	
		DENV	Human TNF	25664975	
		EBOV	Human TNF	27622822	
		HCoV-OC43	Human TNF	27381385	
		HHV-8	Human TNF	27521848	
		SARS-CoV	Human TNF	14592603	
		ZIKV	Human TNF	27916837	Seizures, gastrointestinal symptoms, hypotension, retinopathy, nausea, hair loss, skin reactions.
Ciclopirox	Experimental	HIV-1	Human eIF5A	19825182	Itching, rednes, discoloration of nails.
Cidofovir	Experimental	B19V	Unknown	25446336	
		BKV	Unknown	25790744	Nausea, vomiting, diarrhea, headache, rash, hair loss.
Cimetidine	Experimental	HIV-1	Human HRH2	8950301	
		HSV-2	Human HRH2	2561403	Headache, diarrhea, hallucinations, confusion, depression, arthralgia, breast swelling in men.
Ciprofloxacin	Experimental	BKV	Human parC	25790744	Drowsiness, diarrhea, nausea, anxiety, insomina.
Clarithromycin	Experimental	EBOV	Human KCNH2	27890675	Vomiting, diarrhea, tooth discoloration, headache, rash.
Clemastine	Experimental	RSV	Human HRH1	8729205	Drowsiness, headache, constipation, dry mouth, trouble walking.
Clofarabine	Experimental	HIV-1	HIV-1 RT	27009333	
		HIV-2	HIV-2 RT	25103850	Leukopenia, anemia, infection, vomiting, nausea, diarrhea, rash, fever.
Clomiphene	Experimental	EBOV	Human ESR1	23785035	
		HPV	Human ESR1	8835432	Dizziness, vaginal bleeding, flushing.
Cromoglycate	Experimental	FLUAV	Human KCNMA1	26176755	
		RSV	Human KCNMA1	19107959	Difficulty swollowing, difficulty breathing, rash, allergic reactions.
Cyclosporine	Experimental	HCV	Human PPIA	16382464	
		FLUAV	Human PPIA	16382464	
		ZIKV	Human PPIA	27476412	
		WNV	WNV RNA pol	19451286	Shaking, headache, dizziness, nausea, vomiting, diarrhea.
Cysteamine	Experimental	HIV-1	Human NPY2R	8655998	Vomiting, anorexia, fever, diarrhea, letargy, rash, nausea.
Cytarabine	Experimental	HSV-1	HSV-1 DNA pol	4364763	Fever, nausea, vomiting, loss of bowel or bladder control, problems with vision or hearing, seizures.
Dalbavancin	Experimental	EBOV	Human CYP3A4	26953343	
		MERS-CoV	Human CYP3A4	26953343	
		SARS-CoV	Human CYP3A4	26953343	
		ZIKV	Unknown	27476412	Gastrointestinal toxicity, anema, thrombocytopenia, liver toxicity, infusion site reactions.

Dasatinib	Experimental	BKV	Human ABL1	21088155	Anemia, thrombocytopenia, diarrhea, headache, bleeding, rash, nausea, fluid retention.
		DENV	Human ABL1	23616652	
		HCV	Human ABL1	28674529	
		HIV-1	Human ABL1	26851491	
Decitabine	Experimental	HIV-1	HIV-1 RT	1691617	Headache, gastrointestinal symptoms, nausea, insomnia, drowsiness.
Deferiprone	Experimental	HIV-1	Human eIF5A	19825182	Nausea, vomiting, diarrhea, neutropenia, arthralgia.
Desmethylclozapine	Experimental	DENV	Human DRD2	27572397	Gastrointestinal toxicity, nausea.
Dexamethasone	Experimental	VSV	Human NR3C1	2154755	Insomnia, mood change, acne, sweating, headache, nausea, vomiting.
Dibucaine	Experimental	HEV-A	HEV-A helicase	26856848	Allergic reactions, bleedings, skin irritation.
		HEV-B	HEV-B helicase	26856848	
		HEV-D	HEV-D helicase	26856848	
Dihydroergotamine	Experimental	HSV-1	Human HTR1D	14116560	Changed sense of taste, sore throat, vomiting, nausea, nose bleeds, headache.
Diltiazem	Experimental	HRV-A	Human CACNA1C	16054245	Dizziness, nausea, headache, flushing, fainting, arrhythmia.
Dimethyl fumarate	Experimental	HIV-1	Human RELA	21976775	Flushing, abdominal pain, nausea, diarrhea, vomiting, rash.
Dipyridamole	Experimental	HSV-1	Human PDE10A	11709364	Dizziness, diarrhea, vomiting, headache, rash, arthralgia.
Dorzolamide	Experimental	FLUAV	FLUAV NA	26833677	Cloudy vision, drooping eyelid, flu symptoms, nausea.
Doxycycline	Experimental	CHIKV	Human PADI4	25970853	Diarrhea, abdominal pain, rash, hypoglycemia, hemolytic anemia.
		DENV	Human PADI4	2414227	
		VSV	Human PADI4	26459887	
Dronedarone	Experimental	EBOV	Human ADRA1A	25933611	Diarrhea, nausea, heartburn, weakness, rash.
Enalapril	Experimental	DENV	Human ACE	26321474	Blurred vision, confusion, sweating, diarrhea.
Erlotinib	Experimental	BKV	Human EGFR	21088155	Rash, diarrhea, fatigue, nausea, vomiting, infection.
		EBOV	Human EGFR	28240606	
		HCV	Human EGFR	28240606	
Esomeprazole	Experimental	EBOV	Human ATP4A	26069727	Dizziness, confusion, jerking muscle movements, diarrhea, muscle cramps.
		FLUAV	Human ATP4A	26069727	
		MARV	Human ATP4A	26069727	
Estradiol	Experimental	HCV	Human ER	27885811	Stomach cramps, nausea, breast pain, hair loss, changes in menstrual period.
		FLUAV	Human ER	28557314	
Etodolac	Experimental	HCV	HCV RNA pol	25676325	Diarrhea, constipation, gas, rash, headache, blurred vision.
Ezetimibe	Experimental	HBV	Human NTCP	25034482	Numbness, diarrhea, headache, depression, arthralgia, back pain.
		HCV	Human NTCP	27493122	
		HDV	Human NTCP	24717262	
Famotidine	Experimental	HIV-1	Human HRH2	8950301	Gastrointestinal symptoms, headache, nausea, muscle cramps, arthralgia.
Fenofibrate	Experimental	JEV	Human PPARA	24780919	Severe abdominal pain, nausea, vomiting, flu-like symptmoms, liver toxicity.
Finasteride	Experimental	ZIKV	Human SRD5A2	27919709	Impotence, swelling of breasts, swelling of hands and feet, headache.

Fluoxetine	Experimental	HEV-B	HEV-B helicase	23335743	Drowsiness, nausea, constipation, impotence, insomnia.
		HEV-C	HEV-C helicase	23335743	
		DENV	Human SLC6A4	27572397	
		HCV	Human SLC6A4	25151487	
Fluvastatin	Experimental	DENV	Human HMGCR	27431377	Myalgia, diarrhea, constipation, headache, rash, infection.
		HCV	Human HMGCR	24260365	
		FLUAV	Human HMGCR	24676773	
Formoterol	Experimental	HEV-B	HEV-B helicase	26856848	Allergic reactions, dizziness, tremor, insomnia, chest pain, muscle cramps.
		HRV-B	Human CYP2D6	24998372	
Gefitinib	Experimental	BKV	Human EGFR	21088155	Skin reaction, dry eyes, vomiting diarrhea, bleedings.
		CMV	Human EGFR	18329738	
		VACV	Human EGFR	21094187	
Gemcitabine	Experimental	HEV-B	Human RRM1	26526589	Increased risk of infection, bleedings from gums, nosebleeds, liver toxicity, flu-like symptoms.
		HIV-1	Human RRM1	24120088	
		HRV-A	Human RRM1	28705625	
		HSV-1	Human RRM1	22910914	
		FLUAV	Human RRM1	22910914	
		PV	Human RRM1	27733043	
		SINV	Human RRM1	22910914	
		VACV	Human RRM1	28607950	
		ZIKV	Human RRM1	28049006	
Gemfibrozil	Experimental	HPIV-1	Human PPARA	27915128	Gastrointestinal symptoms, impotence, nausea, drowsiness, myalgia.
Hydroxychloroquine	Experimental	DENV	Human TNF	25321315	Headache, nausea, hair loss, rash, vomiting, spinning sensation.
		HIV-1	Human TNF	8565026	
		ZIKV	Human TNF	28694387	
Hydroxyurea	Experimental	B19V	Human RRM1	28377277	Hair loss, rash, diarrhea, vomiting, headache, weight gain.
		HIV-1	Human RRM1	8852387	
Ibuprofen	Experimental	EBOV	EBOV glycoprotein	28930583	Gastrointestinal symptoms, nausea, nervousness, rash, headache, heartburn.
		HSV-1	Human PTGS2	24768597	
Imatinib	Experimental	BKV	Human BCR/ABL	21088155	Nausea, diarrhea, headache, leg cramps, anemia, risk of infection, bleeding, insomnia.
		HCV	Human BCR/ABL	28674529	
		MERS-CoV	Human BCR/ABL	27466418	
		SARS-CoV	Human BCR/ABL	27466418	
Indomethacin	Experimental	HIV-1	Human PTGS1	7875307	Gas, rectal irritation, drowsiness, rash, blurred vision, headache.
		HSV-1	Human PTGS1	1964373	
		SARS-CoV	Human PTGS1	17302372	
		VSV	Human PTGS1	25856684	
Interferon Beta 1-A	Experimental	HCV	Human IFNAR1	16344588	Flu-like syndrome, allergic reactions.
Itraconazole	Experimental	HEV-B	Human ERG11	25640182	Diarrhea, constipation, nausea, rash, arthralgia, myalgia, headache.
		HRV-B	Human ERG11	25640182	

		HRV-A	Human ERG11	26976677	
		SAFV	Human ERG11	25640182	
Ivermectin	Experimental	CHIKV	Human GABRB3	26752081	
		DENV	Human GABRB3	22535622	
		HIV-1	Human GABRB3	22417684	
		SINV	Human GABRB3	26752081	
		YFV	Human GABRB3	26752081	
		ZIKV	Human GABRB3	27476412	Headache, dizziness, myalgia, diarrhea.
Ketoconazole	Experimental	HSV-1	Human ERG11	3021048	Gastrointestinal symptoms, rash, headache, impotence.
Lansoprazole	Experimental	HRV-B	Human ATP4A	15733557	Diarrhea, stomach pain, nausea, constipation, headache.
Leflunomide	Experimental	BKV	Human DHODH	22461534	
		CMV	Human DHODH	26652481	Gastrointestinal symptoms, nausea, rash, weight loss, back pain.
		EBV	Human DHODH	28574826	
Letermovir	Experimental	HSV-1	HSV-1 terminase	23885997	Diarrhea, nausea, vomiting, peripheral edema, headache, abdominal pain.
Levocetirizine	Experimental	HRV-A	Human HRH1	19110001	Drowsiness, weakness, vomiting, diarrhea, constipation.
		BKV	Human TOP2A	24482066	
Lopinavir	Experimental	ZIKV	ZIKV protease	28712942	
		MERS-CoV	MERS-CoV protease	26198719	Nausea, vomiting, rash, body fat redistribution, headache.
Lovastatin	Experimental	DENV	Human HMGCR	24586275	
		HPIV-1	Human HMGCR	27915128	Stomach pain, nausea, constipation, myalgia, memory loss, insomnia.
		RSV	Human HMGCR	11257039	
Mefloquine	Experimental	EBOV	Human HBA1	27890675	Depression, hallucinations, vomiting, diarrhea, fever.
Memantine	Experimental	FLUAV	FLUAV M2	9588841	
		HCoV-OC43	Human GRIN3A	24227863	
		HIV-1	Human GRIN3A	1707644	Gastrointestinal toxicity, weight loss, bleedings, arthralgia, anxiety.
		ZIKV	Human GRIN3A	28442607	
Metformin	Experimental	HEV-B	Human PRKAB1	24403577	
		DENV	Human PRKAB1	28384260	
		HBV	Human PRKAB1	24164660	
		HHV-8	Human PRKAB1	27147746	Weakness, myalgia, insomnia, dizziness, nausea.
		FLUAV	Human PRKAB1	23831494	
Midostaurin	Experimental	EBV	Human PRKCA	23058855	Flu-like symptoms, trouble breathing, nausea, diarrhea, arthralgia, myalgia.
Mifepristone	Experimental	HIV-1	Human NR1I2	16889838	High fever, severe pelvic pain, vomiting, nausea, diarrhea.
Miltefosine	Experimental	HIV-1	Human ABCB1	22704066	Vomiting, nausea, drowsiness, weakness.
Minocycline	Experimental	DENV	Human PADI4	27396621	
		HIV-1	Human PADI4	24409837	Dizziness, myalgia, nausea, diarrhea, raash.
		WNV	Human PADI4	28878079	
Mitoxantrone	Experimental	HIV-1	Human TOP2A	8460151	

		VACV	Human TOP2A	17928345	Nausea, diarrhea, constipation, heartburn, hair loss, heavy menstrul bleeding.
Montelukast	Experimental	FLUAV	Human CYSLTR1	27094326	Rash, tremor, headache, heartburn, nausea, diarrhea.
Mycophenolic acid	Experimental	HCV	Human IMPDH2	22728816	Diarrhea, stomach pain, nausea, vomiting weight loss, fever, weakness.
		RSV	Human IMPDH2	28775087	
		CHIKV	Human IMPDH2	21070810	
		DENV	Human IMPDH2	12504563	
		ZIKV	Human IMPDH2	27476412	
Nalidixic acid	Experimental	FLUAV	FLUAV NA	26833677	Diarrhea, drowsiness, headache, nausea, vomiting.
Nalmefene	Experimental	RSV	Human OPRM	23222260	Headache, dizziness, fever, somnolence, tremor, confusion, myoclonus.
Naloxone	Experimental	HCV	Human OPRM	22614935	Chest pain, severe nausea and vomiting, seizures, agitation, anxiety.
Naproxen	Experimental	FLUAV	FLUAV NP	23459490	Gastrointestinal symptoms, nausea, heartburn, dizziness.
Nicotine	Experimental	HEV-B	Human CHRNA4	26851533	Headache, constipation, dizziness.
Nilotinib	Experimental	EBOV	Human ABL1	22378924	Diarrhea, constipation, blood in urine and stool, arrhythmia, rash.
Nitazoxanide	Experimental	CHIKV	Human PKR	27742486	Nausea, vomiting, diarrhea,abdominal pain.
		HBV	Human PKR	21553812	
		HIV-1	Human PKR	25303025	
		RV	Human PKR	23926336	
		JEV	Human PKR	24456815	
		MERS-CoV	Human PKR	27095301	
		NoV	Human PKR	21839773	
		RuV	Human PKR	28974385	
		ZIKV	Human PKR	28685770	
Ofloxacin	Experimental	BKV	Human TOP2A	25399012	Diarrhea, seizures, insomnia, fainting, allergic reactions.
Olmesartan medoxomil	Experimental	HEV-B	Human AGTR1	16336207	Headache, runny nose, sore throat, sneezing, difficulty with breathing.
Omeprazole	Experimental	EBOV	Human ATP4A	26069727	Headache, stomach pain, diarrhea, vomiting, gas, cold symptoms.
		FLUAV	Human ATP4A	26069727	
		MARV	Human ATP4A	26069727	
Oritavancin	Experimental	EBOV	Human CYP3A4	26953343	Nausea, vomiting, rash, anemia, arthralgia, hypoglycemia.
		MERS-CoV	Human CYP3A4	26953343	
		SARS-CoV	Human CYP3A4	26953343	
Orlistat	Experimental	DENV	Human PNLIP	28193229	Nausea, vomiting, liver toxicity, nephrotoxicity, allergic reactions.
		HEV-B	Human PNLIP	25680890	
Palbociclib	Experimental	HIV-1	Human CDK4	25036183	Nausea, vomiting, peripheral neuropathy.
		HSV-1	Human CDK4	26542306	
Palonosetron	Experimental	ZIKV	Human HTR3A	27476412	Headache, diarrhea, constipation, hallucinations, confusion.

Pamidronate	Experimental	FLUAV	Human FDPS	26285203	Infusion site reactions, myalgia, headache, nausea, vomiting.
Panretin	Experimental	HHV-8	Human RARA	17374335	Rash, itching, skin inflammation, pain.
Paroxetine mesylate	Experimental	HIV-1	Human SLC6A4	10773489	Nausea, drowsiness, dizziness, insomnia, blurred vision, sweating.
Pentosan polysulfate	Experimental	HEV-A	Human FGF2	22771616	Nose bleed, blood in urine and stool, headache, diarrhea, hair loss, rash.
		HEV-B	Human FGF2	12941917	
		HHV-7	Human FGF2	10480261	
		HIV-1	Human FGF2	7683745	
		FLUAV	Human FGF2	22197247	
Pentoxifylline	Experimental	HIV-1	Human PDE4B	19355959	Gas, vomiting, diarrhea, blurred vision, headache, dizziness.
		JEV	Human PDE4B	18804347	
Pioglitazone	Experimental	HCV	Human PPARG	22412837	Stomach pain, blood in urine, weight gain, chest pain.
Pirlindole	Experimental	HEV-A	HEV-A helicase	26856848	Agitation, tremor, hyperthermia, seizures.
		HEV-B	HEV-B helicase	26856848	
		HEV-D	HEV-D helicase	26856848	
Pitavastatin	Experimental	HCV	Human HMGCR	17928739	Memory problems, myalgia, fever, liver toxicity.
		FLUAV	Human HMGCR	23420457	
Plerixafor	Experimental	HIV-1	Human CXCR4	25888743	Bleedings, gastrointestinal symptoms, insomnia, arthralgia, myalgia, headache.
		HIV-2	Human CXCR4	7562918	
Podofilox	Experimental	CMV	Human TOP2A	27783035	Skin irritation, bleeding, insomnia, nausea, vomiting, pain during sex.
Posaconazole	Experimental	EBOV	Human ERG11	27890675	Gastrointestinal toxicity, insomnia, headache, dizziness.
Pravastatin	Experimental	CMV	Human HMGCR	24976258	Headache, heartburn, memory loss, muscle pain, confusion.
		DENV	Human HMGCR	27431377	
Prochlorperazine	Experimental	DENV	Human DRD2	25028694	Drowsiness, anxiety, missed menstrual period, constipation, weight gain.
Pseudoephedrine	Experimental	RSV	Human SLC6A2	2557947	Anxiety, dizziness, bleedings, arrhythmia.
Pyrimethamine	Experimental	ZIKV	Human DHFR	27476412	Sore throat, rash, flu-like syndrome, blood in urine.
Raloxifene	Experimental	FLUAV	Human ESR1	26684252	Numbness, headache, swelling of hands and legs, vaginal bleeding, flu symptoms.
Ranitidine	Experimental	HIV-1	Human HRH2	8950301	Headache, insomnia, gastrointestinal toxicity, swollen breasts in men.
Rapamycin	Experimental	BKV	Human MTOR	21079551	Gastrointestinal toxicity, anemia, arthralgia, thrombocytopenia.
		HIV-1	Human MTOR	26170311	
Retinoic acid	Experimental	HEV-A	Human RARA	24495389	Back pain, allergic reaction, insomnia, drowsiness, abdominal pain.
Ribavirin	Experimental	LASV	LASV RNA pol	28916737	Heamolytic anemia, worsening of heart disease, teratogenic effect, vision changes, pancreatitis.
Ritonavir	Experimental	MERS-CoV	MERS-CoV protease	26198719	Nausea, diarrhea, vomiting, headache, dizziness.
Roflumilast	Experimental	RSV	Human PDE4D	23936072	Allerginc reactions, weght loss, tremor, diarrhea, flu symptoms.
Romidepsin	Experimental	HIV-1	Human HDAC1	25896701	Nausea, vomiting, hyperglycemia, diarrhea, infection.

Rosiglitazone	Experimental	DENV	Human PPARG	28384260	Fluid retention, swelling, chest pain, bleedings, blurred vision.
Salmeterol	Experimental	DENV	Human LABA	27572397	Headache, nausea, insomnia, vomiting, sweating.
Sertraline	Experimental	ZIKV	Human SLC6A4	27476412	Drowsiness, constipation, insomnia, nausea.
Simvastatin	Experimental	CMV	Human HMGCR	24976258	Worsening of diabetes, rhabdomyolysis, autoimmune myopathy, nephrotoxicity.
		DENV	Human HMGCR	27431377	
		HBV	Human HMGCR	27779671	
		FLUAV	Human HMGCR	25882623	
		PV	Human HMGCR	16824485	
Sitagliptin	Experimental	HCV	Human DPP4	25308370	Runny nose, headache, diarrhea, constipation.
Sofosbuvir	Experimental	ZIKV	ZIKV pol	29352135	Nausea, diarrhea, rash, allergic reactions, drowsiness.
Sorafenib	Experimental	BKV	Human BRAF	21088155	Rash, diarrhea, hand-foot syndrome, hyoertension, hair loss.
		HCV	Human BRAF	25823619	
Sulfasalazine	Experimental	HIV-1	HIV-1 integrase	9021196	Nausea, rash, vomiting, liver toxicity.
Sunitinib	Experimental	BKV	Human PDGFRB	21088155	Diarrhea, nausea, anemia, leukopenia, vomiting, bleeding.
		DENV	Human PDGFRB	28240606	
		HCV	Human PDGFRB	28240606	
Tamoxifen	Experimental	HCV	Human ESR1	17704057	Chills, cofusion, blurred vision, dizziness, arrhythmia.
		HSV-1	Human ESR1	24657267	
Tardiferon	Experimental	HIV-1	Unknown	19355959	Constipation, diarrhea, nausea, vomiting.
Teicoplanin	Experimental	EBOV	Human CYP3A4	26953343	Fever, rash, itching, dizziness, headache, diarrhea.
		MERS-CoV	Human CYP3A4	26953343	
		SARS-CoV	Human CYP3A4	26953343	
Telavancin	Experimental	EBOV	Human CYP3A4	26953343	Diarrhea, drowsiness, fluid retension, confusion.
		MERS-CoV	Human CYP3A4	26953343	
		SARS-CoV	Human CYP3A4	26953343	
Temsirolimus	Experimental	ANDV	Human MTOR	23135723	Rash, nbausea, mouth sores, fluid retention.
Terbinafine	Experimental	HBV	Human SQLE	26456011	Gastrointestinal symptoms, rash, headache, dizziness.
Teriflunomide	Experimental	EBV	Human DHODH	28574826	Liver toxicity, diarrhea, flu symptoms.
Thalidomide	Experimental	HCV	Human TNF	20303772	Flu symptoms, rash, convulsions, anxiety, tremor, insomnia.
Tiotropium	Experimental	HRV-B	Human CHRM3	22362848	Gastrointestinal toxicity, cold symptoms, nose bleed.
		RSV	Human CHRM3	18728351	
Tofacitinib	Experimental	HIV-1	Human JAK1	24419350	Cellulitis, pneumonia, liver toxicity, anemia.
Tolcapone	Experimental	EBOV	Human COMT	28152588	Gzstrointestinal toxicity, unintentional movements, sleeping problems, mental/mood changes, arrhythmia.
Topotecan	Experimental	DENV	Human TOP1	24727241	Flu-like syndrome, constipation, bleeding gums, blood in urine, ulcers in the mouth.
		EBOV	Human TOP1	27127234	

		HCV	Human TOP1	23529728	
		HIV-1	Human TOP1	9145855	
		FLUAV	Human TOP1	27127234	
		JCV	Human TOP1	26935240	
Toremifene	Experimental	EBOV	Human ESR1	23785035	Nausea, sweating, constipation, itching, hair loss, depression.
Tretinoin	Experimental	HHV-8	Human RARA	18505171	Skin, itching, redness, burning, worsening of acne.
Valproic acid	Experimental	WNV	Human ABAT	21106740	Gastrointestinal toxicity, drowsiness, flu-like syndrome, depression, insomnia.
Valsartan	Experimental	HEV-B	Human AGTR1	20392896	Headache, gastrointestinal symptoms, flu symptoms.
Vemurafenib	Experimental	HEV-B	Human BRAF	Unpublished	Headache, skin changes, diarrhea, constipation, hair loss.
		FLUAV	Human MAPK	29312159	
Verapamil	Experimental	CMV	Human CACNA1C	2819886	Dizziness, gastrointestinal symptoms, liver toxicity, fainting,
		EBOV	Human CACNA1C	25722412	
		HRV-A	Human CACNA1C	16054245	
		FLUAV	Human CACNA1C	6743023	
Xantinol	Experimental	HIV-1	Human ADORA1	19355959	Rash, abdominal pain, hypotension, hypoglycemia.
Zuclopenthixol	Experimental	HEV-B	HEV-B helicase	26856848	Headache, fatigue, menstrual problems, anxiety.

Table S2. SaliPhe, SNS-032, obatoclax and gemcitabine differentially affect transcription of immune-related genes in stimulated human PBMC-derived macrophages.

**Drug**	**Stimulus**	**Gene**	**FC, log_2>3, <-3**
No_drug	IAV	*IFNB1*	7,55
		IFNA14	7,39
		IL29	7,32
		IFNA16	7,25
		IFNA2	6,99
		INDO	6,98
		CXCL10	6,93
		RSAD2	6,89
		CCL8	6,81
		IDO1	6,53
		OASL	6,30
		IFNA8	6,27
		IFNA1	6,26
		TNFSF10	6,12
		ISG20	6,06
		IFI44L	5,86
		IFNA7	5,86
		GBP4	5,65

		PTGS2	5,64
		TNF	5,62
		CCL20	5,54
		CCL5	5,49
		GBP5	5,47
		MX2	5,41
		CCL4L1	5,41
		IFITM3	5,32
		ISG15	5,32
		IFIT1	5,17
		IFIT3	5,16
		IFIT2	4,99
		USP18	4,88
		HERC5	4,77
		CCL4L2	4,70
		NEXN	4,68
		IRF7	4,64
		TNFAIP6	4,63
		LOC728835	4,61
		CCL3L1	4,60
		PRIC285	4,56
		IFNA13	4,54
		OAS2	4,51
		OAS3	4,47
		EPSTI1	4,43
		IFI27	4,34
		GCH1	4,33
		HESX1	4,31
		PMAIP1	4,30
		APOBEC3A	4,30
		IFNW1	4,28
		OAS1	4,27
		DDX58	4,25
		GBP1	4,22
		LOC400759	4,17
		MX1	4,16
		IL28B	4,15
		NCF1C	4,13
		IFITM1	4,13
		IL27	4,13
		IFITM2	4,09
		IFNA10	4,07

		NT5C3	4,02
		ADM	4,01
		SAMD9L	3,98
		AXUD1	3,97
		KLF6	3,81
		DUSP19	3,76
		IL6	3,76
		IL28A	3,76
		C5ORF39	3,73
		IFIH1	3,69
		OTUD1	3,68
		HES4	3,68
		LOC387763	3,68
		CCL7	3,67
		CMPK2	3,67
		IL4I1	3,65
		DDX60L	3,64
		GMPR	3,60
		ENPP2	3,59
		LAMP3	3,58
		SAMD9	3,54
		APOL3	3,53
		PDGFRL	3,53
		KIAA1751	3,51
		NCOA7	3,51
		ZC3HAV1	3,50
		CXCL2	3,50
		FAM175A	3,46
		XAF1	3,43
		SP110	3,43
		TNFAIP3	3,39
		IL10RA	3,37
		KIAA1618	3,37
		RTP4	3,35
		NAMPT	3,34
		CD274	3,33
		MXD1	3,32
		PARP9	3,32
		CD69	3,32
		BATF2	3,30
		USP41	3,29
		DDX60	3,28

		STAP1	3,28
		FAP	3,28
		ZBP1	3,25
		HERC6	3,25
		CXCL9	3,21
		IGF2BP3	3,18
		DHX58	3,18
		PARP14	3,16
		IL12A	3,14
		LOC730249	3,13
		IFI35	3,12
		NCF1	3,12
		RABGAP1L	3,10
		IFI44	3,10
		MAP3K8	3,07
		PRDM1	3,05
		EDN1	3,05
		CH25H	3,04
		LOC100128274	3,01
		MS4A14	3,01
		SOCS1	3,00
	dsRNA	*CXCL10*	4,47
		IFIT2	3,44
		IFIT1	3,39
		MX1	3,31
		RSAD2	3,20
		GADD45A	3,10
	IFNa	*IFIT1*	4,40
		IFI44L	4,21
		RSAD2	4,00
		ISG15	3,92
		MX1	3,72
		MX2	3,68
		EPSTI1	3,53
		OAS2	3,18
		ISG20	3,06
	LPS	*CXCL10*	7,14
		IL6	7,05
		INDO	7,05
		IDO1	7,02
		CCL8	6,87
		IL1A	6,81

		CCL20	6,80
		ISG20	6,52
		LOC730249	6,52
		PTGS2	6,49
		CCL3L1	6,19
		RSAD2	5,95
		CSF2	5,94
		TNFAIP6	5,76
		IL1B	5,69
		CXCL2	5,65
		TNIP3	5,55
		IRG1	5,54
		IFI44L	5,46
		GBP4	5,43
		CCL1	5,39
		GBP5	5,37
		TNF	5,37
		LOC728835	5,23
		CCL4L1	5,22
		IFIT1	5,15
		OASL	5,13
		SLAMF1	5,12
		ISG15	5,05
		LRRC50	5,03
		SERPINB2	4,98
		CXCL1	4,97
		GCH1	4,86
		IL23A	4,85
		EDN1	4,84
		IL7R	4,75
		TNFSF10	4,64
		NCF1C	4,61
		IFIT2	4,56
		CCL5	4,54
		NAMPT	4,50
		CCL4L2	4,49
		EPSTI1	4,48
		GBP1	4,48
		STAT4	4,48
		CD38	4,42
		CFB	4,41
		IFI27	4,41

		ADORA2A	4,37
		HES4	4,37
		IL8	4,36
		IFIT3	4,36
		SOD2	4,34
		EBI3	4,33
		IL27	4,23
		ZC3H12A	4,22
		NCF1	4,22
		CD80	4,21
		PDE4B	4,09
		IFITM1	4,05
		IER3	4,05
		IRF7	4,00
		BIRC3	3,99
		MX2	3,99
		ADA	3,99
		MARCKS	3,96
		IFITM3	3,95
		CCR7	3,93
		LAMP3	3,87
		CXCL5	3,86
		MAP3K8	3,82
		HERC5	3,78
		USP18	3,77
		RGS16	3,73
		DUSP1	3,73
		MYO1G	3,72
		APOL3	3,70
		MCOLN2	3,69
		CXCL6	3,66
		MX1	3,65
		PRIC285	3,63
		CCL7	3,61
		HESX1	3,60
		IL1F9	3,59
		G0S2	3,56
		OSM	3,48
		OAS2	3,45
		CMPK2	3,44
		PDGFRL	3,44
		SGPP2	3,36

		IRAK2	3,34
		NLF2	3,34
		SLAMF7	3,33
		SLC25A24	3,33
		LINCR	3,32
		NFKBIZ	3,32
		RNF19B	3,31
		CCL3	3,28
		IGFBP4	3,27
		GREM1	3,27
		OAS3	3,25
		DHX58	3,24
		IFI44	3,22
		NT5C3	3,22
		SERPINE2	3,21
		ANGPTL4	3,21
		OAS1	3,20
		DUSP19	3,20
		LOC400759	3,20
		MIR155HG	3,19
		ANKRD22	3,19
		IL15RA	3,18
		IFITM2	3,16
		STAP1	3,11
		ADM	3,10
		ITGB8	3,10
		TRIM22	3,08
		IFNG	3,08
		IRF1	3,06
		EHD1	3,06
		C15ORF48	3,06
		XAF1	3,05
		PFKFB3	3,05
		SP110	3,05
		CXCL9	3,05
		ZP3	3,05
		GRAMD1A	3,04
		SAMD9	3,02
SaliPhe	IAV	*PLIN2*	3,05
		IFNA2	-6,58
		IFNA14	-6,44
		IL29	-6,36

		IFNA16	-6,03
		IDO1	-5,61
		INDO	-5,60
		IFNA7	-5,54
		IFNA8	-5,52
		IFNA1	-5,41
		IFNB1	-5,34
		PTGS2	-4,75
		CCL5	-4,42
		IFNA13	-4,30
		IFNW1	-4,22
		IL28B	-4,10
		CCL20	-4,08
		IFNA10	-4,02
		IL28A	-3,71
		KLF6	-3,61
		PMAIP1	-3,45
		IL27	-3,44
		CCL3L1	-3,42
		TNF	-3,42
		CCL4L2	-3,35
		IFI27	-3,28
		FAP	-3,25
		HES4	-3,24
		GBP5	-3,20
		ISG20	-3,18
		FAM175A	-3,16
		LOC728835	-3,13
		CXCL9	-3,10
		ENPP2	-3,10
		KIAA1751	-3,09
		MXD1	-3,08
		NCF1C	-3,05
		LOC387763	-3,03
		IL12A	-3,01
		CCL4L1	-3,00
	dsRNA	*GADD45A*	-3,19
SNS-032	No_stim	*C9ORF152*	3,41
		LOC100132715	-3,97
		HCP5	-3,89
		EEF1E1	-3,60
		EXOSC3	-3,44

		SLFN11	-3,33
		GTF2H2B	-3,30
		DIMT1L	-3,30
		STS-1	-3,27
		RGS2	-3,23
		OBFC2A	-3,15
		SRP19	-3,10
		NSMCE4A	-3,09
		SFRS3	-3,08
		GPR34	-3,07
		NFKBIE	-3,04
		MGAT2	-3,02
	IAV	*IFNA16*	-7,19
		IFNA14	-7,16
		IFNA2	-7,00
		IL29	-6,95
		CCL4L1	-6,29
		INDO	-6,25
		IFNA8	-6,11
		IFNA7	-6,11
		LOC728835	-6,02
		IDO1	-6,00
		PTGS2	-5,94
		IFNA1	-5,82
		CCL4L2	-5,63
		CCL20	-5,59
		CCL5	-5,50
		TNF	-5,46
		TNFAIP6	-5,25
		IFNB1	-5,19
		ISG20	-5,19
		CCL3L1	-5,07
		CXCL10	-4,87
		FAM175A	-4,82
		KIAA1618	-4,78
		OASL	-4,75
		OTUD1	-4,65
		IFNA13	-4,63
		KIAA1751	-4,61
		KLF4	-4,58
		CCL7	-4,55
		IL4I1	-4,44

		APOBEC3A	-4,39
		DDX58	-4,38
		IFNW1	-4,38
		F3	-4,36
		GCH1	-4,36
		IL28B	-4,34
		ATF5	-4,33
		TNFAIP3	-4,30
		IFI44L	-4,29
		IFI27	-4,27
		CH25H	-4,27
		HESX1	-4,26
		HES4	-4,15
		MXD1	-4,14
		GBP4	-4,13
		RABGAP1L	-4,10
		MX2	-4,09
		PTGER4	-4,09
		PMAIP1	-4,07
		LOC100132715	-4,06
		IL28A	-4,06
		MCOLN2	-4,05
		IFNA10	-4,05
		IL27	-4,04
		IL6	-4,02
		NCF1C	-3,99
		GEM	-3,99
		ISCA1	-3,98
		EGR1	-3,98
		IRF7	-3,97
		DDX60L	-3,96
		CCL3	-3,96
		CCL8	-3,95
		STAP1	-3,95
		ENPP2	-3,94
		RGS1	-3,94
		CXCL2	-3,92
		RAPGEF2	-3,92
		CDKN2AIPNL	-3,91
		PLSCR1	-3,90
		IER3	-3,88
		TRIM5	-3,88

		PPM1B	-3,87
		PELI1	-3,87
		IFITM1	-3,85
		PSMB9	-3,83
		MIR155HG	-3,83
		LOC100129362	-3,83
		C14ORF85	-3,81
		DNAJB1	-3,81
		ADM	-3,81
		DUSP19	-3,79
		IFIT1	-3,77
		CASP1	-3,77
		PRIC285	-3,76
		HCP5	-3,74
		MS4A14	-3,72
		KLF6	-3,71
		LOC387763	-3,70
		CCL2	-3,70
		CYP27B1	-3,70
		GBP5	-3,69
		C17ORF87	-3,68
		IFI44	-3,68
		IL17RD	-3,68
		C15ORF57	-3,67
		CD40	-3,66
		APOL3	-3,66
		IL8	-3,65
		BTG1	-3,63
		GTF2E2	-3,60
		CITED2	-3,58
		CXCL9	-3,58
		IL1B	-3,56
		EDN1	-3,53
		TNFSF10	-3,52
		ASCL2	-3,52
		NBN	-3,51
		NFKBIE	-3,51
		EBI2	-3,51
		ERICH1	-3,50
		LOC728809	-3,50
		MYLIP	-3,48
		BRDG1	-3,48

		LOC400759	-3,47
		PSTPIP2	-3,46
		PNRC2	-3,45
		GBP3	-3,43
		ZFP36	-3,42
		SOCS1	-3,40
		DDX51	-3,39
		CCRL2	-3,39
		XAF1	-3,39
		HSPA1A	-3,38
		C5ORF39	-3,37
		METTL1	-3,37
		BAMBI	-3,35
		IGF2BP3	-3,34
		INTS3	-3,34
		BMS1P5	-3,34
		LOC100131261	-3,34
		CCNA1	-3,32
		NCF1	-3,32
		SLFN11	-3,32
		GMPR	-3,31
		IL12A	-3,31
		LAMP3	-3,30
		IFITM3	-3,30
		OBFC2A	-3,30
		ZFP36L2	-3,30
		IFI35	-3,29
		KCNH6	-3,29
		PSMD12	-3,29
		C3AR1	-3,29
		EID2B	-3,28
		OAS2	-3,27
		IL10RA	-3,25
		LOC730249	-3,25
		WSB1	-3,24
		FAM26F	-3,24
		SAMD9	-3,23
		CFLAR	-3,23
		TTC39B	-3,22
		DHX58	-3,22
		CD80	-3,22
		EXOSC3	-3,22

		GK	-3,22
		SLAMF7	-3,21
		NAGK	-3,20
		GBP1	-3,19
		PI4K2B	-3,19
		LOC100130516	-3,19
		PARP10	-3,19
		MARCKS	-3,18
		JUN	-3,18
		LOC728620	-3,17
		SMARCA5	-3,17
		PTPN6	-3,17
		GCLC	-3,16
		TRIP4	-3,16
		LOC100128084	-3,16
		TRIM22	-3,16
		LOC100132391	-3,16
		NT5C3	-3,15
		PPP1R15A	-3,15
		NFKBIA	-3,15
		XRN1	-3,15
		APOBEC3G	-3,15
		HS.489254	-3,15
		VAMP5	-3,14
		HPS5	-3,14
		LOC389293	-3,14
		ZBP1	-3,13
		TIA1	-3,13
		DDX60	-3,12
		MGAT2	-3,12
		GPR180	-3,10
		RPAP3	-3,10
		MRPL17	-3,09
		HSPA1B	-3,09
		MLKL	-3,09
		FGD2	-3,09
		HS.133181	-3,09
		HERC4	-3,08
		JAK2	-3,07
		OAS1	-3,07
		NR4A2	-3,07
		CATSPER2	-3,07

		GPR84	-3,07
		RANBP3L	-3,06
		LOC729603	-3,06
		TRIM13	-3,06
		IL7R	-3,05
		NFKBIZ	-3,05
		CCL13	-3,05
		NSMCE4A	-3,05
		EXOSC9	-3,04
		PHF11	-3,02
		XBP1	-3,01
		RCAN1	-3,01
		ZC3H12A	-3,01
		HNRNPD	-3,01
		ISCA1L	-3,01
		FUT4	-3,00
		GDF15	4,06
		TP53BP2	3,06
		TRIM16L	3,04
		AFMID	3,03
	LPS	*LOC730249*	-4,85
		IRG1	-4,64
		RGS16	-3,84
		CXCL6	-3,56
		OSM	-3,41
		CFB	-3,35
		TM4SF1	-3,20
		CXCL5	-3,17
		CCL1	-3,17
		GREM1	-3,14
		ANGPTL4	-3,13
		NCF1C	-3,12
		NLF2	-3,08
Obatoclax	IAV	*AKR1C3*	3,21
		IFNB1	-7,58
		IFNA14	-7,49
		IL29	-7,40
		IFNA16	-7,24
		CCL8	-7,18
		RSAD2	-7,00
		IFNA2	-6,97
		INDO	-6,92

		CXCL10	-6,80
		IDO1	-6,58
		IFNA1	-6,32
		TNFSF10	-6,28
		IFNA8	-6,27
		OASL	-6,12
		ISG20	-6,04
		IFNA7	-5,93
		IFI44L	-5,68
		ISG15	-5,51
		PTGS2	-5,43
		GBP5	-5,38
		USP18	-5,22
		TNF	-5,22
		IFIT3	-5,21
		IFITM3	-5,21
		MX2	-5,05
		CCL20	-5,01
		IFIT1	-4,95
		CCL5	-4,94
		GBP4	-4,92
		GBP1	-4,84
		NEXN	-4,77
		IFIT2	-4,72
		IFNA13	-4,70
		DUSP19	-4,59
		HESX1	-4,59
		PMAIP1	-4,58
		LOC400759	-4,52
		IFI27	-4,48
		CCL7	-4,44
		NT5C3	-4,38
		APOBEC3A	-4,32
		IFNW1	-4,27
		FAM175A	-4,25
		KIAA1751	-4,22
		IFITM1	-4,17
		CCL3L1	-4,15
		DDX58	-4,13
		IL6	-4,12
		IL28B	-4,11
		IFNA10	-4,09

		GCH1	-4,09
		OAS3	-4,08
		ENPP2	-4,06
		C5ORF39	-4,02
		PRIC285	-4,00
		IL27	-3,99
		HES4	-3,99
		IRF7	-3,97
		IL28A	-3,93
		EPSTI1	-3,90
		HERC5	-3,90
		OAS1	-3,85
		IFI44	-3,84
		KLF6	-3,84
		IFITM2	-3,79
		NCF1C	-3,77
		CCL4L2	-3,76
		CD69	-3,72
		CMPK2	-3,72
		KIAA1618	-3,69
		PDGFRL	-3,65
		LOC728835	-3,61
		GMPR	-3,59
		MXD1	-3,58
		OAS2	-3,57
		LOC387763	-3,55
		ADM	-3,54
		CCL4L1	-3,53
		CCL2	-3,53
		XAF1	-3,53
		USP41	-3,52
		STAP1	-3,52
		SAMD9L	-3,47
		SP110	-3,45
		CXCL9	-3,37
		FAP	-3,36
		CH25H	-3,36
		DDX60L	-3,34
		VAMP5	-3,29
		NCOA7	-3,27
		BRDG1	-3,27
		ZC3HAV1	-3,27

		LOC100128274	-3,25
		RPPH1	-3,23
		BATF2	-3,22
		ZBP1	-3,21
		IL12A	-3,21
		IFI35	-3,20
		AXUD1	-3,18
		IL10RA	-3,17
		HERC6	-3,15
		RTP4	-3,15
		SDS	-3,12
		SAMD9	-3,09
		IFNG	-3,07
		CXCL2	-3,07
		LOC730249	-3,07
		IFIH1	-3,06
		TNFAIP6	-3,04
		SLC25A28	-3,04
		IL1RN	-3,03
	dsRNA	*CXCL10*	-4,82
		DUSP1	-4,20
		NFKBIE	-4,19
		PLAU	-4,18
		IFIT1	-4,06
		IFIT3	-3,97
		CEBPA	-3,95
		HPS6	-3,83
		IMP3	-3,82
		CCL3L1	-3,82
		CCRL2	-3,74
		CEBPZ	-3,74
		PNRC2	-3,74
		BTG2	-3,72
		MAFB	-3,68
		IL8	-3,65
		ANKRD57	-3,57
		BTG1	-3,56
		TNFSF14	-3,56
		CCL3	-3,55
		HSPA1B	-3,54
		TNFRSF1A	-3,52
		ZBED5	-3,51

		LOC100131261	-3,50
		XBP1	-3,47
		LOC728835	-3,46
		OKL38	-3,44
		SLC38A2	-3,44
		CCL3L3	-3,44
		RGS2	-3,39
		SERTAD1	-3,39
		FRAT2	-3,35
		RSAD2	-3,32
		GEM	-3,30
		RG9MTD1	-3,29
		C9ORF167	-3,28
		MX1	-3,28
		HERPUD1	-3,28
		C14ORF169	-3,26
		ADM	-3,25
		TRIB1	-3,25
		MGAT2	-3,24
		EXOSC3	-3,24
		ATF3	-3,23
		TSC22D1	-3,19
		PLIN2	-3,19
		HCP5	-3,18
		TNF	-3,17
		IL1B	-3,17
		LOC100132715	-3,17
		TTC4	-3,15
		SLFN11	-3,14
		OBFC2A	-3,14
		PRIC285	-3,13
		HMGCS1	-3,12
		LOC728006	-3,11
		IFIT2	-3,11
		CCL4L2	-3,10
		FADD	-3,10
		IRF9	-3,09
		DNAJB9	-3,09
		SPRED1	-3,07
		HSPA1A	-3,07
		BAZ1A	-3,07
		DUSP5	-3,07

		CCL8	-3,06
		CCDC117	-3,04
Gemcitabine	IAV	*IFNE*	4,47
		IFNE1	3,43
	dsRNA	*CCL3*	-3,41
		CCL3L1	-3,36
		LOC728835	-3,25

Table S3. SaliPhe, SNS-032, obatoclax and gemcitabine differentially affect production of cytokines and growth factors by stimulated human PBMC-derived macrophages.

**Drug**	**Stimulus**	**Protein**	**Up=1/Down=-1**
No_drug	IAV	CCL2	1
		CFD	1
		Angiogenin	1
		PTX3	1
		CCL7	1
		IFNG	1
		CRP	1
		Ang-1	1
		MIF	1
		CXCL10	1
		IL-1ra	1
		CSF2	1
	dsRNA	ApoA1	-1
		CXCL4	-1
		BDNF	-1
	IFNa	ApoA1	-1
		CXCL4	-1
		BDNF	-1
	LPS	CXCL1	1
		IL-6	1
		CCL2	1
		PTX3	1
		IFNG	1
		CRP	1
		Ang-1	1
		IL-1a	1
		TNF-a	1
		CCL3/CCL4	1
		IL-1ra	1
		CSF2	1

		CD147	1
SaliPhe	IAV	VDBP	-1
		ApoA1	-1
		CCL2	-1
		CFD	-1
		Angiogenin	-1
		PTX3	-1
		CCL7	-1
		IFNG	-1
		CRP	-1
		Ang-1	-1
		MIF	-1
		CXCL10	-1
		IL-1ra	-1
		CSF2	-1
	IFNa	LCN2	1
		PDGF-AA	1
		CCL2	1
		CD31	1
		EGF	1
		uPAR	1
SNS-032	IAV	CXCL5	-1
		ApoA1	-1
		CHI3L1	-1
		VDBP	-1
		LCN2	-1
		PDGF-AA	-1
		CCL2	-1
		CFD	-1
		Angiogenin	-1
		CD31	-1
		PTX3	-1
		CCL7	-1
		IFNG	-1
		CRP	-1
		Ang-1	-1
		CXCL4	-1
		MIF	-1
		BDNF	-1
		CCL5	-1
		TSP-1	-1
		RBP-4	-1

		CXCL10	-1
		IL-1ra	-1
		EGF	-1
		uPAR	-1
		CD147	-1
		MMP-9	-1
	dsRNA	CXCL5	-1
		OPN	-1
		LCN2	1
	IFNa	CXCL5	-1
		OPN	-1
		EGF	1
	LPS	CXCL1	-1
		CXCL5	-1
		OPN	-1
		ApoA1	-1
		IL-6	-1
		LCN2	-1
		VDBP	-1
		CD31	-1
		CCL2	-1
		PTX3	-1
		IFNG	-1
		CRP	-1
		Ang-1	-1
		IL-1ra	-1
		BDNF	-1
		TNF-a	-1
		CCL3/CCL4	-1
		RBP-4	-1
		EGF	-1
		IL-1ra	-1
		uPAR	-1
		CSF2	-1
		CD147	-1
Obatoclax	No_stim	CXCL5	-1
		CHI3L1	1
		LCN2	1
		CCL2	1
		CCL3/CCL4	1
		RETN	1
	IAV	CXCL5	-1

		ApoA1	-1
		LCN2	-1
		PDGF-AA	-1
		VDBP	-1
		Angiogenin	-1
		CFD	-1
		CCL2	-1
		CD31	-1
		PTX3	-1
		CCL7	-1
		IFNG	-1
		CRP	-1
		Ang-1	-1
		CXCL4	-1
		MIF	-1
		BDNF	-1
		CCL5	-1
		TSP-1	-1
		RBP-4	-1
		EGF	-1
		IL-1ra	-1
		CXCL10	-1
		uPAR	-1
		CSF2	-1
		MMP-9	-1
	dsRNA	CXCL5	-1
		CHI3L1	1
		LCN2	1
		VDBP	1
		PDGF-AA	1
		CD31	1
		CCL2	1
		CXCL4	1
		CCL3/CCL4	1
	IFNa	CXCL5	-1
		CHI3L1	1
		LCN2	1
		VDBP	1
		PDGF-AA	1
		CD31	1
		CCL2	1
		CCL3/CCL4	1

		EGF	1
		CXCL10	1
		RETN	1
	LPS	CXCL1	-1
		ApoA1	-1
		LCN2	-1
		IL-6	-1
		VDBP	-1
		CCL2	-1
		PTX3	-1
		IFNG	-1
		CRP	-1
		Ang-1	-1
		CXCL4	-1
		IL-1a	-1
		BDNF	-1
		CCL3/CCL4	-1
		IL-1ra	-1
		CSF2	-1
		CD147	-1
		RETN	1
Gemcitabine	IAV	ApoA1	-1
		LCN2	-1
		IL-6	-1
		VDBP	-1
		Angiogenin	-1
		CFD	-1
		CCL2	-1
		CD31	-1
		PTX3	-1
		CCL7	-1
		IFNG	-1
		CRP	-1
		Ang-1	-1
		CXCL4	-1
		MIF	-1
		CCL5	-1
		TSP-1	-1
		BDNF	-1
		RBP-4	-1
		EGF	-1
		IL-1ra	-1

		uPAR	-1
		CD147	-1
		MMP-9	-1
	IFNa	CXCL4	-1
		RBP-4	-1
	LPS	ApoA1	-1
		IL-6	-1
		LCN2	-1
		VDBP	-1
		CD31	-1
		CXCL4	-1
		TNF-a	-1
		CCL3/CCL4	-1
		RBP-4	-1
		CSF2	-1
		MMP-9	-1

Table S4. SaliPhe, SNS-032, obatoclax and gemcitabine, differentially affect production of several polar metabolites by stimulated PBMC-derived macrophages.

**Drug**	**Stimulus**	**Metabolite**	**FC, log2>1.5, <-1.5**
No_drug	IAV	SAM	2,78
		Inosine	2,40
		Creatine	2,33
		Adenosine	1,82
		Cotinine	1,71
		TMNO	-2,70
	dsRNA	Cotinine	1,74
		Creatine	1,66
		Aspartate	-2,74
	IFNa	SAM	2,12
		Aspartate	-2,87
	LPS	SAM	2,64
		SAH	1,75
		Aspartate	-2,42
SaliPhe	No_stim	Cotinine	1,89
	IAV	TMNO	1,64
		Inosine	-2,04
	dsRNA	TMNO	-2,49
	LPS	Glycocholic Acid	2,29
		Creatine	1,59
		SAM	-3,80
		Uracil	-1,91
SNS-032	No_stim	SAM	3,49

		Inosine	2,92
		SAH	2,11
		Sucrose	1,92
		Adenosine	1,75
		Chenodeoxycholic acid	1,68
		Creatine	1,65
		TMNO	-2,12
	IAV	TMNO	1,64
		SAM	-1,72
	dsRNA	Inosine	3,13
		Creatine	-1,94
	IFNa	Inosine	2,64
		SAH	2,17
		Aspartate	1,90
		Adenosine	1,56
		Cotinine	-2,28
	LPS	Inosine	2,39
		SAM	-3,28
		TMNO	-1,61
Obatoclax	No_stim	Adenosine	3,19
		Succinate	2,03
		Cotinine	1,84
		Uracil	1,81
	IAV	TMNO	2,52
		Succinate	1,77
		Aspartate	1,62
		SAM	-2,03
		Inosine	-1,61
	dsRNA	SAM	2,82
		Aspartate	2,44
		Adenosine	1,92
	IFNa	Aspartate	2,92
		Adenosine	1,91
		Succinate	1,61
	LPS	Aspartate	2,73
		Succinate	1,63
		Propionylcarnitine	1,54
		SAM	-2,35
		SAH	-2,16
Gemcitabine	No_stim	Ornithine	1,86
		Isovalerylcarnitine	1,58
		SAM	-2,42

		Histidine	-2,30
		1-methylhistamine	-2,01
		Uracil	-1,68
	IAV	TMNO	2,39
		SAM	-1,80
	dsRNA	TMNO	-2,11
		Uracil	-1,99
	IFNa	SAM	-2,82
		Uracil	-2,67
	LPS	SAH	1,89
		SAM	1,55
		Uracil	-2,19
